# Comprehensive spectroscopy and photocatalytic activity analysis of TiO_2_-Pt systems under LED irradiation

**DOI:** 10.1038/s41598-024-64748-4

**Published:** 2024-06-15

**Authors:** Adam Kubiak

**Affiliations:** Faculty of Chemistry, Adam Mickiewicz University, Poznan, Uniwersytetu Poznanskiego 8, PL-61614 Poznan, Poland

**Keywords:** Heterogeneous catalysis, Photocatalysis, Photochemistry, Semiconductors, Nanoparticles, Synthesis and processing, Photocatalysis

## Abstract

This study presents a thorough spectroscopic analysis of TiO_2_-Pt systems under LED irradiation, with a focus on elucidating the photodeposition process of Pt nanoparticles onto TiO_2_ surfaces. The methodology leverages an innovative LED photoreactor tailored to a specific spectral range, enabling precise characterization of the excitation spectrum of TiO_2_-Pt composites. Through the identification of Pt precursor species and their excitation under LED-UV light, a photodeposition mechanism is proposed involving concurrent excitation of both the TiO_2_ semiconductor and the H_2_PtCl_6_ precursor. The LED photoreactors are employed to scrutinize the excitation profile of TiO_2_-Pt materials, revealing that the incorporation of Pt nanoparticles does not expand TiO_2_'s absorption spectrum. Furthermore, UV-A exposure in the absence of Pt did not induce the formation of surface defects, underscoring the lack of visible light activity in TiO_2_-Pt systems. Spectroscopic analyses, complemented by naproxen photooxidation experiments, indicate the absence of a significant plasmonic effect in Pt nanoparticles within the experimental framework. Mass spectroscopy results corroborate the presence of distinct naproxen degradation pathways, suggesting minimal influence from photocatalyst properties. This research provides a detailed spectroscopic insight into TiO_2_-Pt photocatalysis, enriching the knowledge of photocatalytic materials in LED lighting.

## Introduction

In the era of sustainable development, a critical parameter is striving to minimize the environmental impact, especially during the design phase of new processes^1^. However, the pressing need for the utilization of photocatalysts active in visible light may engender concerns regarding the actual performance of the material within a given spectral range. It is imperative to highlight that these novel photocatalytic materials are frequently evaluated using outdated exposure systems reliant on optical filters or similar solutions. Consequently, there is a potential for a misleading assessment of the material's activity under visible light^2^. A pivotal solution in this realm is the advancement of photoreactor engineering. Regrettably, in the realm of photocatalytic water treatment, the scientific community predominantly favors material development over reactor engineering at this juncture^3^. Yet, within the context of the ongoing energy revolution, which emphasizes the utilization of light-emitting diodes (LEDs) as efficient light sources, it becomes evident that the future trajectory of the field of photochemistry is illuminated^4^. It's worth highlighting the undeniable advantages of LEDs over conventional light sources, including the absence of toxic mercury found in traditional lighting^5^. Moreover, LEDs can operate at lower voltages and are constructed from durable materials^6,7^. Additionally, when compared to the conventional cylindrical shape of mercury vapor lamps, LEDs can be approximated as point sources, allowing for more flexible designs^8,9^. It's essential to emphasize that apart from the evident benefits concerning their impact on the natural environment, LED diodes also contribute to increased efficiency in photo-oxidation processes compared to mercury lamps, as reported by Kubiak et al.^10^. Furthermore, studies by Bertagna Silva et al.^11^ and Jo and Tayade^12^ have shown that LEDs not only reduce energy costs but also enhance device geometry. However, in addition to enhancing the efficiency of photooxidative processes, LEDs can serve as a novel tool to elucidate mechanisms that have not yet been delineated or to provoke skepticism within the scientific community.

One such process that continues to generate uncertainty, despite extensive research, is the manner in which metallic nanoparticles (NPs) interact with semiconductors^13–15^. These interactions have sparked significant interest in various fields, including energy, sensors, biomedicine, catalysis, and energy storage, owing to the highly adjustable optical, electronic, magnetic, sensing, and catalytic properties of these nanoparticles^16–19^. By exercising precise control over the design of NPs, one can modify and optimize their inherent properties, thereby enhancing their suitability for various device applications. Nevertheless, questions persist about the specific mechanisms involved, particularly when considering the impact of different metallic nanoparticles like gold, silver, and platinum on semiconductors^20–22^. While many researchers attribute the interaction of gold and silver nanoparticles with visible light to localized surface plasmon resonance (LSPR), the mechanism is less clear when it comes to platinum nanoparticles. First and foremost, it's important to acknowledge a certain contradiction that is evident in the available scientific literature. Specifically, Qi et al.^23^ have demonstrated an enhancement in absorption within the visible-light spectrum, attributed to the localized surface plasmon resonance (LSPR) absorption of Pt nanoparticles (NPs). However, on the contrary, Kunwar et al.^24^ have reported that Pt NPs do not exhibit a strong LSPR response akin to that observed in Ag and Au nanoparticles. Furthermore, Jian Zhao et al.^25^ have illustrated that plasmonic material such as platinum can initially interact with neighboring electroactive materials and subsequently play a pivotal role in the transfer of hot carriers. These observations find support in existing scientific reports. Jiao He et al.^26^ have reported that Pt@UiO-66(Zr) synthesized materials did not effectively harness visible light for photoactivity. However, its efficiency could be significantly enhanced by adsorbing dyes, leading to the sensitization of TiO_2_. A similar effect was documented by Hayat Khan et al.^27^, who noted that Pt^0^ nanoparticles loaded onto the synthesized Pt-TiO_2_/WO_3_ catalyst could serve as an electron sink, capturing, storing, and releasing electrons diffusing from excited semiconductors to generate superoxide anions, all while increasing electron–hole separation. On the other hand, it's important to consider scientific findings from sources such as Li et al.^28^, who suggest the feasibility of plasmonic excitation of Pt on a bismuth oxide substrate. Furthermore, the utilization of LSPR for exciting platinum nanoparticles was also demonstrated in the Fe_2_O_3_@Pt@FeS system, as reported by Hyerim Park et al.^29^. Similar scientific studies have also been documented involving the use of titanium dioxide as a semiconductor. Hongfei Shi et al.^30^ reported the potential for plasmonic excitation of platinum in Pt/POMs/TiO_2_ systems.

The study delves into platinum photodeposition on TiO_2_ substrates, utilizing an advanced LED photoreactor with specific spectral output for detailed excitation analysis of TiO_2_-Pt composites. Spectroscopic methodologies elucidate TiO_2_-Pt interactions under varied illumination, revealing that Pt nanoparticle integration doesn't broaden TiO_2_'s excitation spectrum. This key insight, from extensive spectroscopic analysis, is vital for comprehending photocatalytic dynamics sans attributing activity enhancement to excitation range expansion. The research contributes nuanced perspectives on TiO_2_-Pt photocatalysts' spectroscopic characteristics under LED irradiation.

## Materials and method

### Materials

Anatase (Sigma-Aldrich, 99,7%), AEROXIDE® P25 (Thermo Scientific, ACS reagent), chloroplatinic acid hexahydrate (Thermo Scientific, ACS reagent, ≥ 37.50% Pt basis), methanol (Sigma-Aldrich, ACS reagent, ≥ 99.8%), naproxen (NPX) (Acros, > 98%). All reagents were of analytical grade and used without any further purification. The water used in all experiments was deionized.

### Synthesis of TiO_2_-Pt materials

TiO_2_-Pt materials were produced using the photodeposition technique with a typical LED solution emitting light in the wavelength range of 360–400 nm. The procedure entailed dispersing 1 g of TiO_2_ (either anatase or P25) in a 50 mL water:methanol solution (1:1 v/v). Subsequently, 300 µL of chloroplatinic acid hydrate (0.1 wt% solution) was added to achieve a Pt/TiO_2_ ratio of 1 wt%. The mixture was sealed and purged with argon for 30 min. Subsequently, the suspension was irradiated for 30 min with LED light. This irradiation caused a color change from orange (indicating the presence of the platinum precursor) to gray, confirming the successful photoreduction of Pt(IV) to metallic platinum nanoparticles. Subsequently, the samples were filtered and washed with water to eliminate potential impurities and any remaining unreduced precursor residues. The samples were dried at 60 °C for 6 h. These samples, synthesized using anatase and P25, were labeled as A-Pt and P25-Pt, respectively. Finally, reference samples (A_365nm and P25_365nm) have been synthesized following exactly the same procedure, except for the addition of platinum precursor.

### Characterization of fabricated materials

X-ray diffraction (XRD) assessment was conducted using a D8 Advance diffractometer from Bruker in Germany. This instrument utilized Cu Kα radiation (λ = 1.5418 Å), with nickel filtration. The data was collected through step-scanning, with increments of Δ2θ = 0.05°, spanning an angular range between 20° and 80°.

The porous structure parameters of exfoliated graphite/Pt were determined using a 3FLEX surface characterization analyzer (Micromeritics Instrument Co., USA). The Brunauer–Emmett–Teller (BET) method, based on low-temperature N_2_ sorption, was utilized for the analysis. The surface area was determined using the multipoint BET method, utilizing adsorption data in the *p/p*_*0*_ relative pressure range of 0.05–0.30.

Transmission electron microscopy (TEM) analysis was conducted using the FEI TECNAI G2 F20 electron microscope. The microscope operated at an acceleration voltage of 200 kV and featured a Gatan CCD camera capable of high-resolution imaging. To prepare specimens for high-resolution TEM (HR-TEM) analysis, a small amount of material was sonicated in 2-propanol and then suspended onto a copper grid with a holey carbon film. Micrographs were captured following solvent evaporation, covering the entire sample area to ensure a comprehensive and statistically representative mapping of the studied materials.

The platinum content was assessed employing an Epsilon4 EDXRF spectrometer (PANalytical, United Kingdom) that operates on energy dispersion (EDXRF) principles. This spectrometer is furnished with an X-ray tube featuring an Ag 5W anode and a 50 kV generator with 500 uA current. It includes a set of six measurement filters and a high-resolution SDD semiconductor detector cooled by the Peltier effect.

Agilent's 5500 atomic force microscope was utilized to perform AFM measurements in intermittent contact mode under ambient conditions. The material being tested was obtained from a solution and applied to the surface of mica. Prior to application, the substrate surface was cleaned through mechanical stripping. The test material was then applied onto the substrate via spin coating. Scanning was done using the Allin One cantilever type with a resonance frequency of approximately 150 kHz, manufactured by BudgetSensors. The collected data was analyzed using WSxM and Gwyddion software.

The concentration of metal ions in the aqueous solutions was assessed through the utilization of Atomic Absorption Spectroscopy (AAS) ContrAA300 (Analytik Jena, Germany), employing a wavelength of 265.9 nm. The determination of Pt(IV) concentration involved applying a calibration curve represented by the equation y = (0.0000627 + 0.0015604·x)/(1 + 0.0017031·x).

For the evaluation of platinum content on the surface of the TiO_2_ materials, an ICP-OES analysis was conducted, following a methodology outlined elsewhere. The Pt loading was ascertained by digesting 15 mg of the TiO_2_ material in aqua regia (3:1 HCl:HNO_3_), allowing the solution to stabilize overnight. Subsequently, Varian 710-ES ICP optical emission spectrometer (Varian, USA) was employed for the analysis after diluting one milliliter of it with 13 cm^3^ of deionized water.

X-ray Photoelectron Spectroscopy (XPS) experiments were conducted using a Specs UHV spectrometer from SPECS in Germany, which included a charge neutralizer. The binding energies were calibrated using the C 1 s peak at 284.8 eV as a reference.

The light-absorption characteristics of all materials were assessed via diffuse reflectance spectroscopy (DRS) within the wavelength range of 200–800 nm. To determine the bandgap energy of the samples, we employed a method involving (F(R)·E)^0.5^ plotted against photon energy (E), where F(R) represents the Kubelka–Munk function, which is directly related to the absorption of radiation. These measurements were carried out using a Thermo Scientific Evolution 220 spectrophotometer (Waltham, USA), equipped with a PIN-757 integrating sphere, and BaSO_4_ was used as a reference material.

For emission and excitation measurements, a spectrofluorometer (Fluorolog version-3, Horiba, Japan) was employed, with a 450 W high-pressure xenon arc lamp serving as the excitation source. The photoluminescence excitation occurred at a wavelength of λ_excitation_ = 320 nm, and emission spectra were recorded at room temperature, utilizing a spectral resolution of 2 nm with a slit width of 2 mm. The excitation spectra were recorded at λ_emission_ = 440 nm at room temperature, employing a spectral resolution of 2 nm with a slit width of 2 mm.

### Photocatalytic activity

#### Home-made LED-based light source

For the photocatalytic tests, an LED photoreactor was prepared to operate within a narrow wavelength range of 10 nm. The maximum LED wavelengths used were 365, 380, 400, 410, 430, and 450 nm. A single high-power diode was mounted on an aluminum heat sink (FISCHER ELEKTRONIK, Germany) using thermally conductive glue (AG TermoGlue, Poland). The LEDs were then connected to an LED driver (MeanWell, Taiwan). Finally, the radiation intensity was measured, amounting to 20 mW/cm^2^ in each case.

#### Photooxidation tests

Each 0.1 L aqueous suspension subjected to irradiation contained a consistent 1 g L^−1^ concentration of photocatalyst and an initial NPX concentration of 20 mg L^−1^. Achieving adsorption/desorption equilibrium of the substrate on the photocatalyst surface was accomplished by placing the suspension in a light-occluded container and employing magnetic stirring (IKA Werke GmbH, Germany) for 30 min before commencing irradiation. Stirring was maintained throughout the photocatalytic experiments while exposing the reaction mixture to radiation using the custom-designed LED apparatus described earlier. At various time intervals during the experiments, 3 mL aliquots of the suspension were extracted from the photoreactor and filtered through a syringe filter (Macherey–Nagel, Germany). The supernatant was then subjected to spectrophotometric analysis at 330 nm, which corresponds to the maximum absorption wavelength of NPX, utilizing a Thermo Scientific Evolution 220 spectrophotometer. To evaluate the photocatalytic effectiveness of the materials under investigation, the degradation yield (W) was calculated using the following formula:

1 $$W\left(\%\right)=\left(1-\frac{{C}_{t}}{{C}_{0}}\right)\cdot 100\%$$

where C_0_ and C_t_ represent the initial NPX amount and the concentration of NPX determined upon a fixed irradiation time, respectively.

#### Kinetic study

The kinetics of naproxen photooxidation were assessed using a pseudo-first-order kinetic model. This model postulates that the degradation rate is directly proportional to the surface coverage (θ) of NPX, expressed as follows:

2 $$r=\frac{dC}{dt}=k\theta =\frac{kK{C}_{0}}{1+K{C}_{0}+{K}_{s}{C}_{s}}$$

Here, *k* represents the reaction rate constant, 'θ' denotes the surface coverage by naproxen, *K* and *K*_*s*_ are the adsorption coefficients for NPX and water, respectively, C_0_ stands for the initial concentration of NPX, and C_s_ represents the concentration of water. The concentration of water, C_s_ remains nearly constant and is significantly higher than the concentration of NPX. Consequently, we can express Eq. (3) in the following form:

3 $$ln\frac{{C}_{t}}{{C}_{0}}=-{k}_{1}t$$

In Eq. (3), *k*_*1*_ signifies the first-order rate constant, and *t* is the time of irradiation.

#### Verification of the degradation mechanism using scavengers

The study aimed to comprehend the role of charge carriers and reactive oxygen species in the photocatalytic reaction, providing insights into the mechanism of degradation of organic contaminants in the presence of the synthesized TiO_2_-Pt systems. To evaluate the photocatalytic activity, the method described in Sect. Photooxidation tests was employed, with the introduction of scavenger solutions in appropriate quantities. The concentrations of the scavenger solutions were adjusted to achieve a level of 20 mg L^−1 ^for NPX. Specifically, ammonium oxalate was selected as the scavenger for holes (h^+^), AgNO_3_ for electrons (e^-^), tert-butyl alcohol for free hydroxyl radicals (^*^OH), and benzoquinone for superoxide radical anions (^*^O_2_^–^).

#### Tests using wastewater from a municipal sewage treatment plant

Photocatalytic tests were conducted using wastewater from an operational sewage treatment plant in Chwałków, Greater Poland Voivodeship, Poland, in April 2024. This facility manages sewage originating from Środa Wielkopolska, a municipality with a population of over 50,000 residents. The tests involved utilizing sewage from the secondary settling tank. Prior to each experiment, samples of sewage were collected, with the naproxen concentration set at 20 mg/L. The photocatalytic examination followed the protocol outlined in Sect.  Photooxidation tests.

#### MS measurement

To better identify the byproducts resulting from the photocatalytic oxidation of NPX using variously prepared TiO_2_-Pt samples, we conducted MS measurements. The ESI–MS spectra were acquired using a Bruker amaZon SL ion trap instrument (Germany), which was equipped with an electrospray ion source operating in infusion mode. The sample solution was introduced into the ionization source at a flow rate of 5 μL min^-1^ via a syringe pump. The instrument operated in the *enhanced resolution mode* within a mass range of 50–30000 m*/z*, scanning at a rate of 8100 m*/z* per second. The capillary voltage was set to + 4.5 kV, with an endplate offset of -500 V. The ion source temperature was maintained at 80 °C, while the desolvation temperature was set to 250 °C. Nitrogen served as the cone gas, and helium as the desolvating gas, with flow rates of 800 L h^-1^ and 50 L h^-1^, respectively. The mass spectrometer was operated in both positive and negative ionization modes.

## Results and discussion

### Characterization of Pt precursor solution

To gain a deeper understanding of the enhancement of TiO_2_-Pt photocatalytic activity through the photodeposition of Pt nanoparticles on the TiO_2_ surface, we commenced our investigation by first recording the absorption and excitation spectra of the initial Pt precursor solutions. The collected data are presented at Figure S1 in Supplementary Materials.

**Figure 1:**
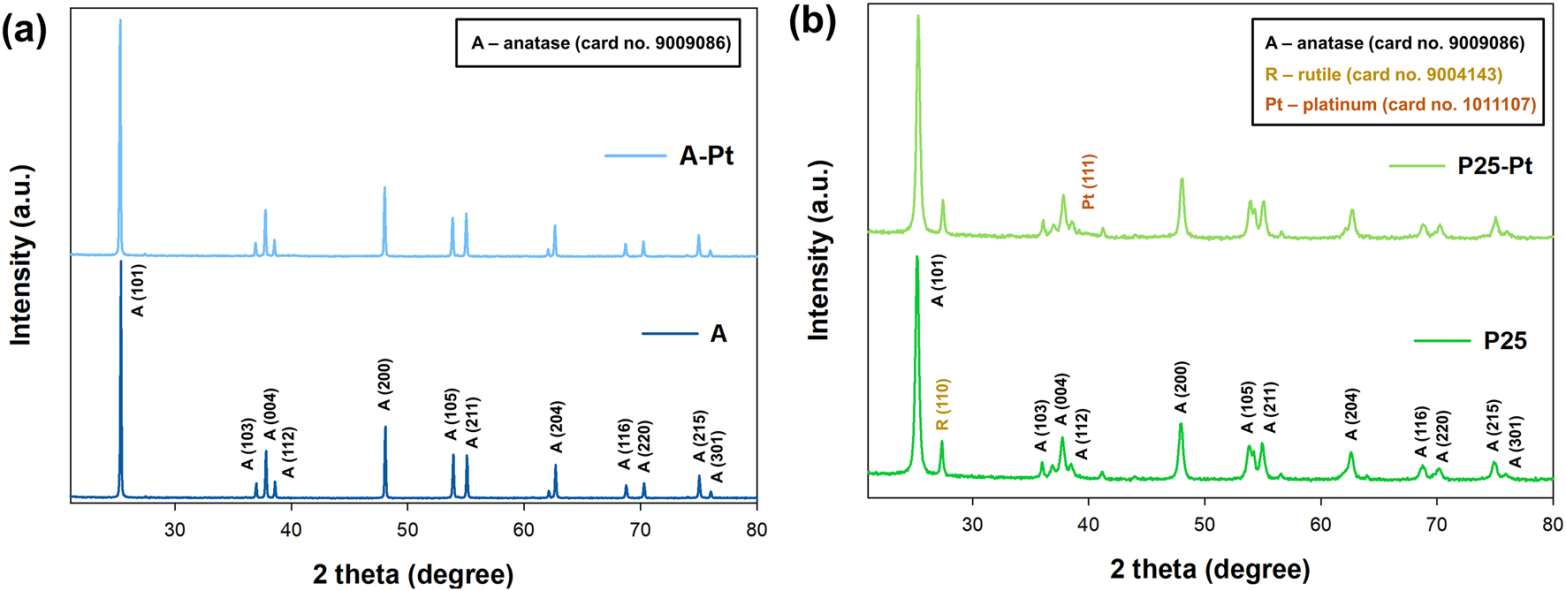
The XRD pattern for TiO_2_-Pt materials synthesized using (**a**) anatase, (**b**) P25.

Based on the data obtained, the primary absorption peak for H_2_PtCl_6_ species is observed at a wavelength of 262 nm, a finding also corroborated by Burgetha and Kisch^31^. However, the absorption spectrum can exhibit additional residual bands within the UV-A and blue light range. Due to this observation, an excitation spectrum was recorded at an emission wavelength of 520 nm. The presented excitation spectrum in Fig. 1b shows the effective excitation of the selected platinum precursor, across a broad range of wavelengths, extending from UV-B to blue light. Furthermore, there were noticeable residual excitation bands in the range of 500–530 nm. Nevertheless, we were able to distinguish two distinct primary excitation peaks at 365 and 450 nm.

Consequently, the process of Pt photodeposition seems to rely on the concurrent activation of both TiO_2_ and platinum precursor species, such as H_2_PtCl_6_, which can adhere to the surface of titania^32,33^. In this context, the deposition of Pt nanoparticles likely occurs through a combination of mechanisms, primarily involving the transfer of photoexcited electrons from the TiO_2_ conduction band to adsorbed Pt(IV) species. Furthermore, there may also be a direct photoreduction of [PtCl_6_]^2−^ complexes upon their photoactivation^34,35^.

### Characterization of TiO_2_-Pt materials

Figure 1 shows the X-ray diffraction patterns of the bare TiO_2_ matrix and samples after the Pt photodeposition process. In the case of the samples where anatase was used as the starting material, the reflections at 2θ = 25.2, 36.7, 37.5, 38.7, 47.8, 53.6, 55, 62.6, 68.8, 70.4, and 75.1° correspond to the (101), (103), (004), (112), (200), (105), (211), (204), (116), (220), and (215) planes of the anatase phase (card no. 9009086)^36,37^. In the case of the A-Pt series, no additional peaks were observed, either from other crystallographic forms of TiO_2_ or related to Pt nanoparticles. For the series using P25 as the starting material, an additional peak at 2θ = 27.6 was observed, corresponding to the (110) plane of rutile (card no. 9004143)^38^. Moreover, for the P25-Pt sample, a weak diffraction peak at 2θ = 40.1, corresponding to the (111) plane, can be seen, indicating the presence of metallic platinum (card no. 1011107)^39^. A close-up of the XRD pattern confirming the additional peak originating from platinum is shown in Figure S2 in the Supplementary Materials.

The presence of Pt NPs diffraction peaks in the diffraction pattern for P25-Pt suggests the effective deposition of nanocrystalline Pt particles on the material's surface, possibly organized in clusters large enough to give rise to characteristic diffraction signals of appreciable intensity. Importantly, in both analyzed series, no shifts in the TiO_2_ pattern peaks were observed upon Pt loading, indicating that Pt is not incorporated into the lattice of TiO_2_ but is rather deposited onto the oxide surface.

To assess the influence of the photodeposition process induced by LED light on the crystal structure of TiO_2_, we conducted an experiment without adding a platinum precursor. The obtained XRD patterns of the reference A_365 nm and P25_365 nm samples showed no significant differences (see Figure S3 in the Supplementary Materials). This suggests that the LED light source alone does not impact the crystal structure of TiO_2_.

Taking into account the relationship between crystallinity and texture structure, a low-temperature nitrogen sorption analysis was performed to further characterize the materials. The nitrogen adsorption–desorption isotherms for the TiO_2_-Pt materials are depicted in Figure S4 of the Supplementary Materials.

The nitrogen adsorption isotherms exhibited characteristics typical of type IV, featuring a hysteresis loop at higher pressure values^40^. Across the analyzed nanomaterials, the hysteresis loop displayed type H3 behavior, indicative of slit-shaped pores formed by aggregates of plate-like particles^41^. Notably, this effect is accentuated by the process of platinum photodeposition on the anatase surface, particularly in P25, where the amount of adsorbed nitrogen increased more than threefold post-Pt deposition. However, it should be noted that the specific surface area remained relatively stable; only a slight increase in BET area (approximately 1–2%) was observed. The most significant modifications were seen in pore parameters, such as pore volume and diameter. For instance, P25 displayed a more than twofold increase in pore volume. Similarly, anatase, despite its smaller surface area, also exhibited notable changes in pore structure. These findings underscore that while the photodeposition of platinum on the TiO_2_ surface modifies the porous structure, it does not significantly expand the surface area.

**Figure 2:**
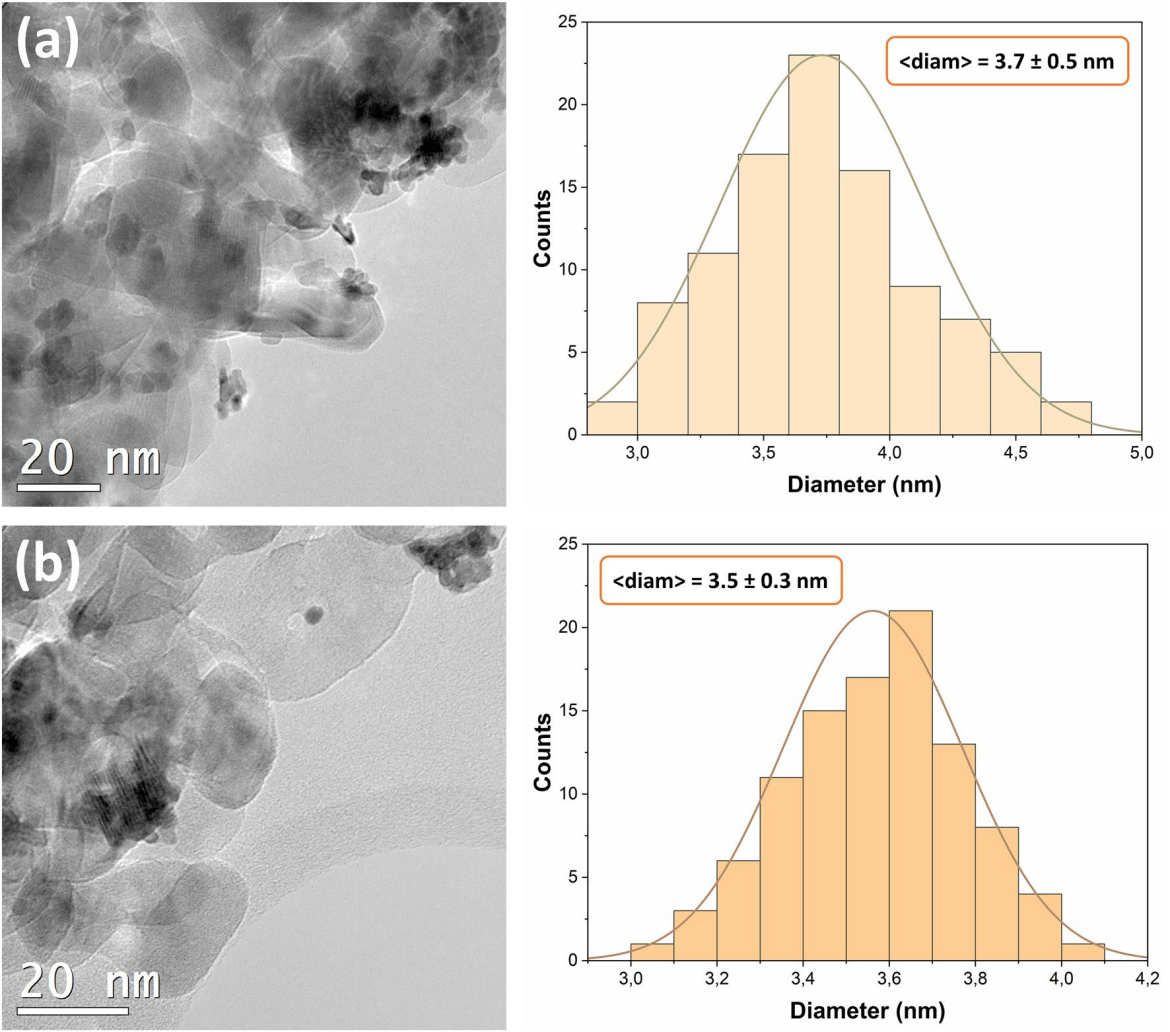
TEM images and PSD analyses for TiO_2_-Pt materials synthesized using (**a**) anatase, (**b**) P25.

To characterize the morphology of photodeposited Pt nanoparticles on widely-used TiO_2_ photocatalysts, high-resolution transmission electron microscopy (TEM) was performed. The noble metal's particle size distribution (PSD) was analyzed through the acquired TEM images (see Fig. 2). Notably, the shape and dispersion of the Pt nanoparticles anchored to the TiO_2_ surface were similar across the different materials analyzed. The use of a designated LED solution for photodeposition facilitated a uniform dispersion of Pt nanoparticles, which had average sizes of approximately 3.7 nm ± 0.5 nm and 3.5 nm ± 0.3 nm for anatase and P25, respectively. Thus, our observations clearly indicate that the distribution of Pt nanoparticles is independent of the titanium dioxide matrix utilized.

**Figure 3:**
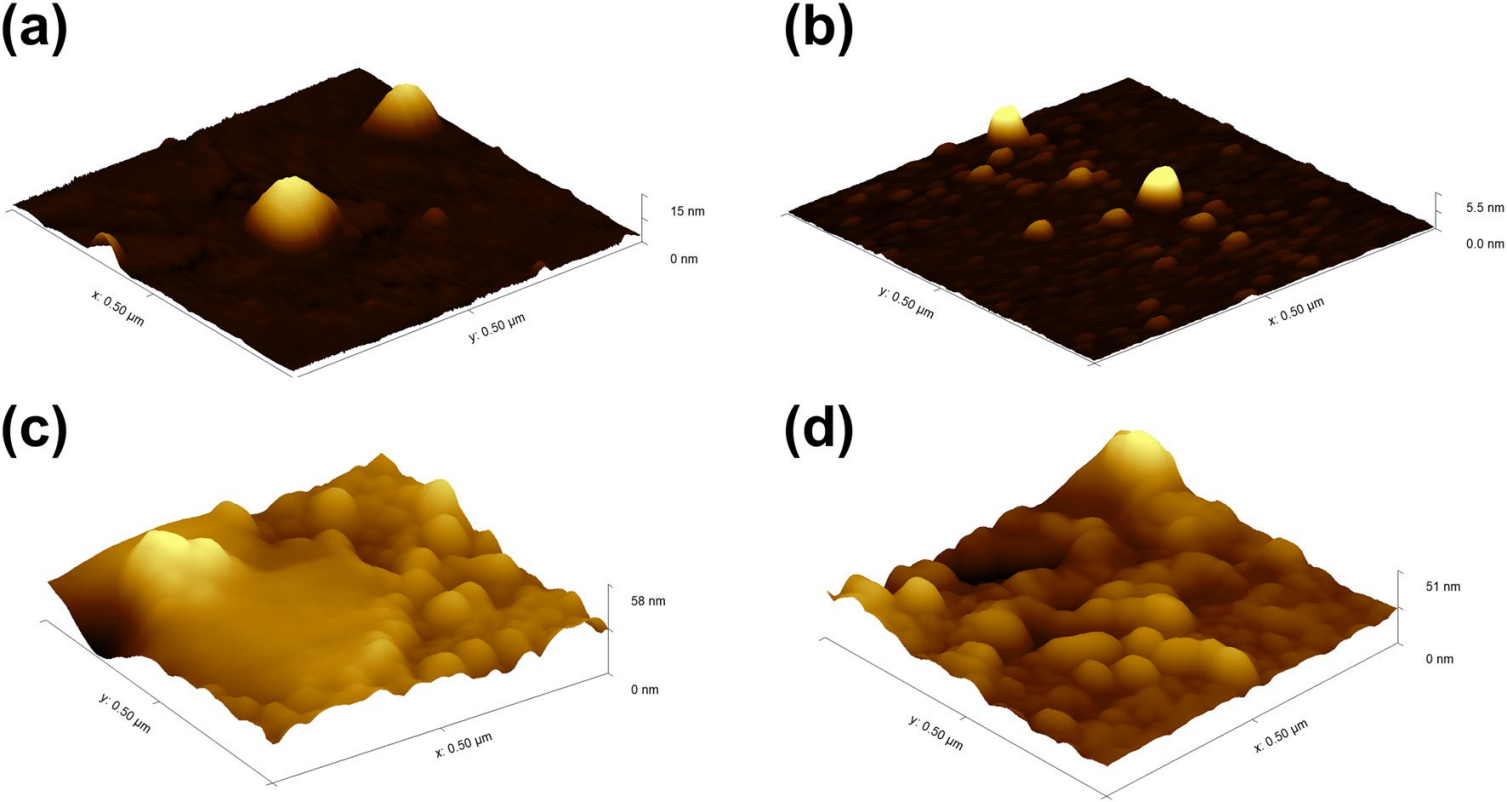
The AFM images for (**a**) A, (**b**) P25, (**c**) A-Pt, and (**d**) P25-Pt.

The atomic force microscope (AFM) was utilized to examine the surface morphology before and after the photodeposition process, as illustrated in Fig. 3.

Each figure represents a close-up view of a 0.5 μm square on the surface of the analyzed samples. For the unmodified samples, both anatase (Fig. 3a) and P25 (Fig. 3b) displayed a lower maximum height along the z-axis, ranging from 5–15 nm. The higher height observed in the case of anatase may be attributed to the presence of individual aggregates, which were also evident in TEM images. Following the platinum photodeposition process, a notable increase in height along the *z*-axis is observed, measuring 58 nm for anatase and 51 nm for P25, respectively. These findings corroborate the efficacy of the platinum photodeposition process.

EDXRF, ICP-OES and AAS analyses were employed to confirm the platinum loading in the synthesized TiO_2_-Pt materials. Based on the obtained data, it has been confirmed that the synthesized materials contain platinum in an amount of 1 wt.% on the TiO_2_ surface. This observed loading closely aligns with the anticipated theoretical value. Furthermore, AAS results indicate that the majority of the platinum content is effectively deposited on the TiO_2_ surface. The obtained results are presented in Table S1 in the Supplementary Materials. Moreover, the SEM/EDX analysis revealed a uniform distribution of platinum nanoparticles across the surface of the TiO_2_ matrices. The findings are detailed in Figure S5 of the Supplementary Materials.

**Figure 4:**
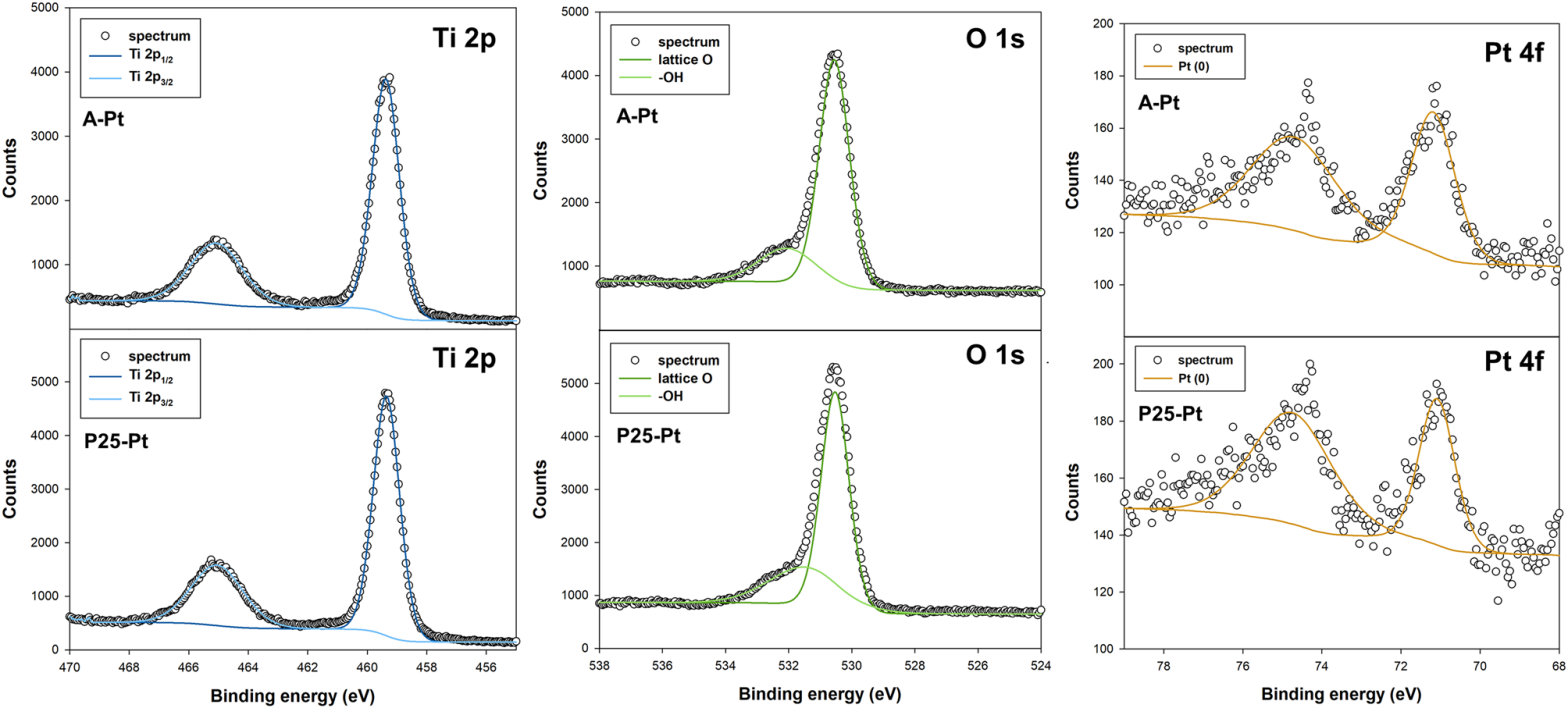
The results of XPS analysis for TiO_2_-Pt materials synthesized using different TiO_2_ matrix.

X-ray photoelectron spectroscopy was used to determine the surface composition and oxidation states of elements present in TiO_2_-Pt systems. Figure 4 presents high-resolution XPS spectra for the Ti 2p, O 1 s, and Pt 4f. binding energy (BE) regions of the studied TiO_2_-Pt materials.

Two peaks were detected in the Ti 2p region, with binding energies centered at 458.5 eV and 464.2 eV, representing Ti 2p_3/2_ and Ti 2p_1/2_, respectively^42,43^. The energy difference of 5.7 eV between these peaks signifies the existence of Ti^4+^ in the sample^44^. The fact that there is no shift in energy for the Ti 2p peaks confirms that the introduction of platinum occurs solely as a surface modification, without the integration of Pt into the TiO_2_ lattice structure. The O 1 s region on the spectra of analyzed materials shows a double peak, which after deconvolution may be resolved to two types of oxygen atoms, the lattice oxygen (530.5 eV), and adsorbed surface -OH groups (532.3 eV)^45,46^. The Pt 4f. region spectrum was analyzed and fitted with a doublet, with a separation of 3.33 eV between the f_7/2_ and f_5/2_ peaks^47^. The mainline 4f_7/2_ peak was observed at a binding energy of 71.1 eV, indicating the presence of metallic platinum on the surface^48,49^.

To investigate the effect of LED light irradiation on the structure of TiO_2_, we conducted an experiment without the addition of a platinum precursor. Figure S6 (see in Supplementary Materials) shows the high-resolution XPS spectra for the Ti 2p region before and after LED light exposure. In all analyzed spectra, two characteristic peaks with binding energies centered at 459.3 eV and 465 eV are observed, corresponding to Ti 2p_3/2_ and Ti 2p_1/2_, respectively^42^. The splitting between Ti 2p_1/2_ and Ti 2p_3/2_ is 5.7 eV, indicating the presence of Ti^4+^ in its normal state^43^. The absence of Ti^3+^ states suggests that UV-LED light irradiation does not modify the structure of TiO_2_ and does not create surface defects.

### Photocatalytic ability

Diffuse reflection spectroscopy (DRS) was used to examine the optical characteristics of the photocatalysts that were synthesized. The collected spectra are shown in Figure S7 in Supplementary Materials.

The photodeposition process is well-known for its inability to modify the material's band gap^50,51^. It's important to note that platinum, being non-incorporated into the titanium oxide lattice structure, is not considered a doping process. However, at the interface between the platinum nanoparticles (Pt NPs) and anatase, a phenomenon referred to as band-bending occurs^52^. Nevertheless, this phenomenon does not impact the intrinsic band gap values. Consequently, both anatase and anatase-Pt samples exhibit a band gap energy of 3.2 eV, while P25 and P25-Pt samples maintain a band gap of 3.0 eV^53,54^. The observed alterations in the diffuse reflectance absorption spectra following the photodeposition of Pt NPs can be attributed to the significant plasmonic absorption characteristics of these nanoparticles, which overlap with the anatase spectrum.

**Figure 5:**
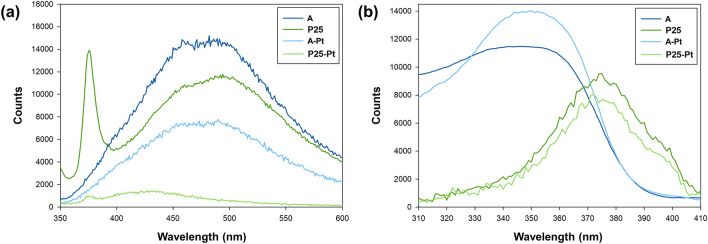
(**a**) emission and (**b**) excitation spectra for TiO_2_-Pt materials synthesized using anatase and P25 as the TiO_2_ matrix.

The emission and excitation spectra of the TiO_2_-Pt materials produced were acquired using fluorescence spectroscopy. The obtained findings are presented in Fig. 5.

Photoluminescence (PL) in semiconductors such as TiO_2_ typically arises from the recombination of photo-generated carriers (Fig. 5a). In our study, we observed a distinct luminescent band centered at approximately 500 nm for anatase samples, consistent with existing literature, including observations from P25 samples which also exhibit a center around ∼500 nm, reflecting pure anatase emissions^55,56^. The presence of a 380 nm peak in the P25 spectrum is likely a reflection of the 330 nm exciting light off the CaF_2_ window, entering the PL spectrometer at an oblique angle, a phenomenon similarly reported by Ma et al.^57^. Notably, luminescence efficiency is reduced in P25, primarily due to the anatase–rutile heterojunction, which effectively enhances charge carrier separation through favorable band alignment that facilitates electron migration and prevents recombination. Upon depositing Pt onto TiO_2_, a significant reduction in the intensity of the 500 nm luminescent band was noted, indicative of decreased charge carrier recombination. This suppression can be attributed to the migration of excited electrons from TiO_2_ to Pt nanoparticles, thus effectively quenching the electron–hole recombination process^58,59^. These results underscore the role of Pt photodeposition in enhancing luminescence quenching, highlighting its potential to improve photocatalytic efficiency.

Due to the potential impact of continuous UV light exposure on modifying the surface of titania by introducing new surface defects, we employed photoluminescence spectroscopy to examine the reference anatase and P25 samples exposed to LED light, without the presence of Pt precursor species (see Figure S8 in Supplementary Materials). As previously reported by Stevanowic et al.^60^, a photoluminescence band at approximately 500 nm serves as a reliable indicator of UV-induced changes in the band structure. Our data unequivocally shows that the unmodified materials exposed to UV-LED (365 nm) exhibited consistent luminescence intensity, affirming prior findings that indicate the absence of surface defects on unaltered materials.

The analysis of the obtained excitation spectra (Fig. 5b) for the unmodified TiO_2_ matrix confirms previous research findings. In the case of anatase, a single excitation band was observed, spanning a wide UV range from 310 to 390 nm. Conversely, for P25, there was a noticeable blue shift, falling within the 330–410 nm range^61^. This shift can be attributed to the existence of a type II heterojunction between anatase and rutile, a phenomenon corroborated by Jiang et al.^62^. However, it's crucial to emphasize that when examining samples subjected to the photodeposition process, no significant deviations in the excitation spectrum are apparent. There are no discernible shifts, and the band edges closely resemble those of the unmodified samples. This discovery contradicts previous observations by scientists, suggesting that after Pt photodeposition on the TiO_2_ surface, these samples also exhibit activity in the visible light spectrum^63^. Chen et al.^64^ have highlighted the visible light activity of TiO_2_-Pt materials. It's important to note that even though the Pt NPs modification is confined to the surface, the authors observed a reduction in the bandgap energy. The capacity to utilize visible light was also confirmed by Baiju Vijayan et al.^65^, who synthesized TiO_2_-Pt nanotubes using the hydrothermal method. However, the authors pointed out that EPR spectroscopy data demonstrates the presence of oxygen vacancies and associated Ti^3+^ ions on the nanotube surface during synthesis, which facilitate the absorption of visible light and the excitation of electrons from these surface defect sites to the conduction band of titania. Nonetheless, He et al.^66^, in their study focusing on the mechanistic analysis of p-phenol oxidation using the I-TiO_2_/Pt material, arrived at similar findings to those described in our research. They highlighted that platinum modification could enhance the separation of electron–hole pairs while minimally affecting the excitation properties of the TiO_2_ matrix. However, the approach that indicates the possibility of plasmonic excitation of Pt nanoparticles is still popular in the available scientific literature. This approach was proposed by Sim et al.^67^, who used graphene oxide/Pt–TiO_2_ nanotube arrays for visible-light-driven photocatalytic reduction of CO_2_.

**Figure 6:**
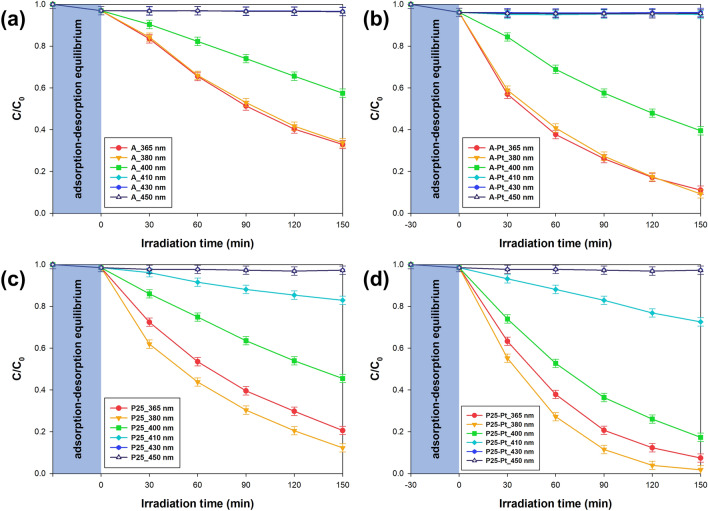
Efficiency of naproxen photocatalytic degradation efficiency achieved with varying LED wavelengths for (**a**) anatase (**b**) anatase-Pt, (**c**) P25, and (**d**) P25-Pt series materials.

Therefore, considering the variations in the existing knowledge documented in the scientific literature, the primary objective of this study was to investigate the photocatalytic potential of TiO_2_-Pt materials across different wavelength spectrums. To accomplish this, a specialized LED photoreactor capable of operating within a range of wavelengths was designed. As a representative test particle for assessing photoactivity, naproxen, a commonly encountered pollutant in real wastewater, was chosen. The obtained photocatalytic curves are collected in Fig. 6. Additionally, the Supplementary Materials include selected UV–Vis spectra from the photocatalytic tests performed (see Figures S9 and S10).

In the initial stage of our analysis, we examined the results for materials in series A (Fig. 6a) and A-Pt (Fig. 6b). In both cases, the highest efficiency in removing naproxen occurred at wavelengths of 365 and 380 nm. This finding carries particular significance in light of the excitation spectrum presented earlier. Notably, the primary absorption band for both material series under investigation spans a wide range from 310 to 370 nm. Beyond the 370 nm threshold, a marked decrease in absorption becomes evident. Nonetheless, it is worth highlighting that the efficiency of naproxen removal using a 380 nm LED remains comparable. Several contributing factors may explain this phenomenon. Firstly, it is essential to acknowledge the exceptionally high intensity of the LEDs. As a result, despite reduced absorption, a substantial photon flux is delivered to the material, enabling effective excitation and subsequent removal of the targeted pollutant. Furthermore, our previous research has consistently demonstrated the successful excitation of anatase-based materials, thereby substantiating earlier conjectures. Lastly, it is important to note that LEDs, while often misconstrued as emitting monochromatic light, actually emit light within a narrow wavelength range. Therefore, LEDs with a peak emission at 380 nm encompass a spectral range of 375–385 nm, closely aligning with the absorption maximum of anatase. Consequently, taking these factors into consideration, the efficacy of anatase excitation within the 375–380 nm range should be regarded with confidence, emphasizing the innovative insights derived from our comprehensive analysis. Shifting the LED wavelength even further to its peak at 400 nm significantly diminishes the effectiveness of naproxen removal in both examined cases. This decrease can primarily be attributed to the reduced absorption of anatase within the studied wavelength spectrum of 390–405 nm. When it comes to A-Pt_400nm samples, the removal efficiency of the targeted pollutant reaches approximately 60%, whereas for bare A_400nm, it's at 40%. Nevertheless, changing the LED wavelength to blue light (410, 430, and 450 nm) results in a complete loss of photoactivity. The test confirms that anatase samples containing platinum nanoparticles on the surface do not absorb visible light. This investigation conclusively demonstrates that the photodeposition of platinum does not broaden the anatase absorption band because the decline in photocatalytic efficiency occurs within the same wavelength ranges for both A-Pt and unmodified anatase samples.

When analyzing the results for both the P25 (Fig. 6c) and P25-Pt (Fig. 6d) series, it's important to highlight their consistency with our prior findings. The highest efficiency in removing naproxen was achieved at an LED wavelength of 380 nm, in line with the previously presented excitation spectrum of these materials. According to scientific literature, P25 exhibits an absorption shift toward blue light due to the presence of two crystalline phases of titanium dioxide—anatase and rutile. This shift enhances the separation of charge carriers through the heterojunction mechanism, as confirmed by the photoluminescence spectra. Consequently, using light with a maximum wavelength of 400 nm also yielded satisfactory naproxen removal results, particularly in the P25-Pt sample series, where photooxidative efficiency exceeded 80% after 150 min of exposure. However, further shifting the wavelength towards blue light (λ_max_ = 410 nm) significantly reduced naproxen removal efficiency, primarily due to residual material absorption within this range. Changing the LED wavelength to 430 nm and 450 nm resulted in the complete loss of photoactivity. It's crucial to emphasize that the demonstrated activity of P25 samples in visible light is not due to the presence of platinum on the material's surface. Ohtani et al.^68^ have confirmed that the heterojunction between the two polymorphic forms of TiO_2_ (anatase/rutile) allows residual absorption above 400 nm. Therefore, this study affirms that the platinum photodeposition process does not alter the excitation range of the tested material. The observed enhancement in photocatalysis is a result of the improved separation of charge carriers.

The revised Langmuir–Hinshelwood model offers a suitable framework for elucidating the degradation mechanism, aligning with the surface reaction concept frequently examined in existing literature^69–71^.

**Figure 7:**
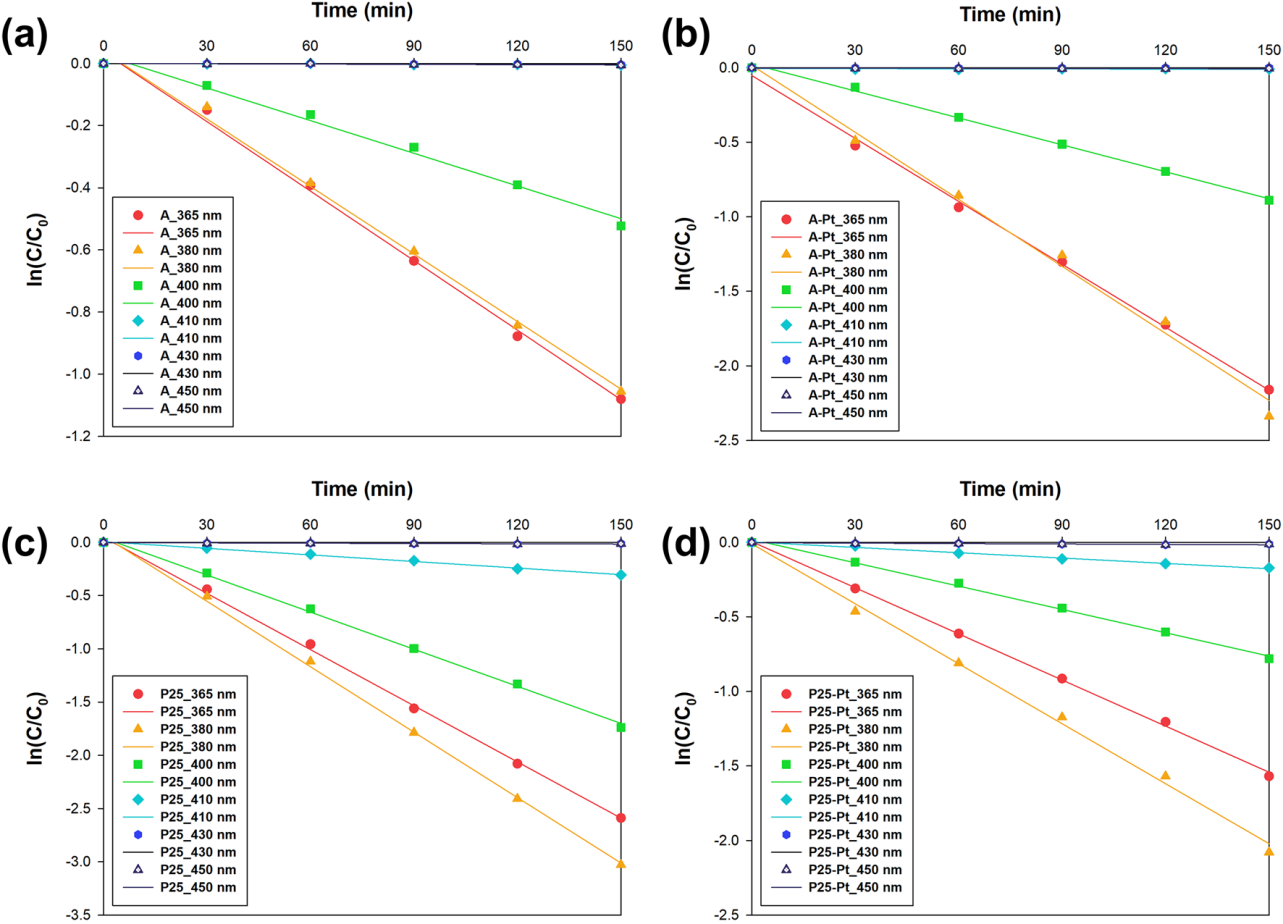
First-order plots of the photooxidation of naproxen using (**a**) A, (**b**) A-Pt, (**c**) P25, and (**d**) P25-Pt photocatalysts.

To establish the rate constant parameter, the sorption process was intentionally omitted from the calculations, as depicted in Fig. 7. According to Eqs. (2) and (3), the apparent values of parameter k_1_ for each catalyst were computed by analyzing the slope of the ln C_t_/C_0_ vs. time plot. The calculated values are presented in Table S2, which can be found in the Supplementary Materials for comparison.

The obtained results indicate that the photooxidative capacity of the prepared materials is highly dependent on the wavelength used in the photocatalytic process. Similarly, kinetic studies have revealed that the starting material—whether it is anatase or P25—significantly influences the resulting photocatalytic activity. As a result, among a series of materials using anatase as the starting material, the A-Pt_365nm material exhibits the highest reaction rate constant at 0.0145 min^−1^. In the case of the P25 series, the P25-Pt_380nm sample records the highest reaction rate constant of 0.0199 min^−1^. Irrespective of the measurement series analyzed, a shift in the wavelength of the LED used in the naproxen photooxidation process leads to a reduction in the reaction rate constant. This reduction is primarily attributed to diminished absorption of longer wavelength radiation, both in the case of anatase and P25. Consequently, it is crucial to consider the excitation spectra of the titanium dioxide matrices employed when planning photocatalytic tests with TiO_2_-Pt materials. It should be noted that platinum nanoparticles do not enhance the absorption of visible light but do facilitate improved separation of charge carriers, as demonstrated in the PL spectra, in line with our previous observations.

Figure S11, as presented in the Supplementary Materials, illustrates the results of experiments where various scavengers were introduced into a solution of NPX within TiO_2_-Pt photocatalysts. These scavengers were utilized to intercept photo-induced electrons, holes, and crucial reactive oxygen species, notably hydroxyl radicals (^*^OH) and superoxide radicals (^*^O_2_^–^). The photooxidation efficiency of NPX showed a decrease of approximately 20% following the inclusion of electron and hole scavengers such as ammonium oxalate and silver nitrate. This slight reduction in NPX degradation efficiency could be attributed to improved charge carrier separation, likely facilitated by the incorporation of yttrium ions into the TiO_2_ structure, as supported by Hu et al.^72^, and Benavente et al.^73^, and further substantiated through photoluminescence spectral analysis. Moreover, the introduction of tert-butanol, targeting hydroxyl radicals, resulted in a significant decline in photodegradation efficiency by approximately 51% for the tested materials, highlighting the crucial role of ^*^OH radicals in the photo-oxidation process. Conversely, the targeting of superoxide radical anions (^*^O_2_^-^) had a noticeable impact on NPX degradation efficiency, resulting in a decrease of 26% and 20% for materials anatase_Pt and P25_Pt, respectively, thereby emphasizing the significant contribution of these radicals to the photocatalytic degradation pathway. These findings suggest that the generation of reactive oxygen species (ROS), particularly hydroxyl and superoxide radicals, predominantly directs the photocatalytic processes in these aqueous systems, as indicated by Lopis et al.^74^, and Kusiak-Nejman et al.^75^.

Naproxen, a persistent organic compound with two aromatic rings, can experience degradation through the involvement of oxidizing species such as ^*^O^2−^, and ^*^OH, and generated upon the excitation of TiO_2_-Pt photocatalysts^76^. Hence, to assess whether the LED wavelength employed during photooxidation processes can impact the degradation pathway, ESI mass spectrometry measurements were carried out.

In the TiO_2_-Pt photocatalytic system employed in this study, the degradation of naproxen can be explained as a decarboxylation process. Initially, hydroxyl radicals attack the methyl position of the naphthalene ring, leading to decarboxylation and the formation of intermediates TP1 (*m/z* 185) and TP2 (*m/z* 201)^77^. In an alternative pathway, the oxidation of Naproxen begins with the action of the superoxide radical, targeting carbon atoms with the highest positive charge, resulting in the creation of carbon-centered radical species. These radicals subsequently undergo decarboxylation and are transformed by the superoxide radical into TP1 (*m/z* 185) and TP3 (*m/z* 223) intermediates^78^. Additionally, decarboxylation can be initiated by an ROS attack, resulting in the formation of the TP4 (*m/z* 158) intermediate^79^. Ultimately, ring-opening reactions take place, followed by further oxidation (TP5, TP6), leading to the conversion into CO_2_ and H_2_O^78,79^. A Table S3, displaying the detected intermediate patterns, can be found in the Supplementary Materials. Furthermore, Table S4 presents selected mass spectra for reference.

Recent studies on the photocatalytic degradation of naproxen illustrate the complex interactions of chemical reactions that enhance environmental management. Jung et al.^80^ identified multiple intermediates using a hybrid TiO_2_ photocatalyst system, emphasizing the critical roles of hydroxylation and demethylation by hydroxyl radicals. Marotta et al.^81^ explored the kinetics of naproxen's photodegradation under UV light, specifically examining how oxygen influences degradation rates and intermediate distributions. Furthermore, Jallouli et al.^82^ confirmed the effectiveness of TiO_2_-UV photocatalysis in decomposing naproxen, with a focus on identifying by-products and evaluating their ecological risks. Collectively, these studies highlight the potential of photocatalytic processes in addressing pharmaceutical pollutants in water.

Our investigations reveal that platinum nanoparticles deposited on the surface of titanium dioxide, both in the anatase form and the commercially available P25, do not expand the excitation spectrum as initially hypothesized. This observation clarifies that under UV light exposure, electrons in the valence band of TiO_2_ are excited, transitioning to the conduction band^83,84^. This process results in the generation of electron–hole pairs, critical for photocatalytic activity.

Significantly, the role of Pt nanoparticles as electron traps due to their substantial work function is confirmed^85^. This trapping of electrons enhances the separation efficiency of electron–hole pairs, thereby enhancing the photocatalytic process. The trapped electrons on the Pt nanoparticles are capable of reacting with water to produce hydroxyl radicals, a key component in the oxidation of naproxen^86^.

Moreover, the valence band holes may react with surface hydroxyl groups to further produce hydroxide radicals. These radicals are crucial for the degradation of organic pollutants such as naproxen. However, under irradiation with visible light (λ > 420 nm), the TiO_2_ matrix remains inactive due to the lack of surface defects like oxygen vacancies, which are typically required for absorption in the visible range.

Additionally, photodeposited platinum nanoparticles do not exhibit localized surface plasmon resonance strong enough to generate a potential difference capable of driving the photooxidation of naproxen. Consequently, the application of blue light does not alter the concentration of naproxen, aligning with our findings that the physicochemical properties of TiO_2_ solely influence the reaction rate constant and do not dictate the degradation pathway of naproxen.

In this comparative study of Pt's role across different TiO_2_ systems, distinct interactions between Pt nanoparticles and the anatase and P25 phases of TiO_2_ were observed. The photocatalytic degradation of naproxen was employed as a functional probe to elucidate these interactions.

In the anatase-Pt system, a significant enhancement of photocatalytic activity, compared to pure anatase, was facilitated by the presence of Pt nanoparticles. It was indicated by spectroscopic analyses that a more efficient separation of charge carriers within anatase is facilitated by Pt. A marked reduction in the recombination rate of photo-generated electron–hole pairs, attributed to the effective trapping capabilities of Pt nanoparticles, was revealed by photoluminescence studies. These findings were corroborated by bandgap measurements, which showed no significant change, thus confirming that the electronic structure of anatase was not altered but the dynamics of charge carriers were improved.

Conversely, in the P25 system, which inherently contains both anatase and rutile phases, a different impact was exhibited by the addition of Pt nanoparticles. Although an enhancement in photocatalytic activity was observed, it was less pronounced than in the anatase-Pt system. This difference can be attributed to the pre-existing heterojunction between anatase and rutile in P25, which already facilitates efficient charge separation. The role of Pt in this context seems to enhance existing pathways for electron transfer rather than creating new dynamics, as evidenced by the lesser reduction in photoluminescence intensity compared to the anatase-Pt system.

To quantify these effects, reaction rate constants for TiO_2_ matrices during and after the platinum photodeposition process were measured. In the case of anatase, it was found that the reaction kinetic constant increased more than twofold. However, for P25, this increase was 1.8-fold, demonstrating a more significant enhancement in photocatalytic activity in the anatase-Pt system due to the synergistic effect of Pt. These findings suggest that while photocatalytic activity is enhanced across both TiO_2_ systems by Pt nanoparticles, the mechanisms of interaction and the extent of enhancement vary markedly. In anatase, primarily as an efficient electron trap, Pt acts by significantly reducing recombination, whereas in P25, the natural heterojunction properties are augmented without dramatically altering the photocatalytic pathway. These insights are deemed crucial for tailoring Pt-TiO_2_ composites for specific environmental or industrial applications requiring optimized photocatalytic efficiencies.

**Figure 8:**
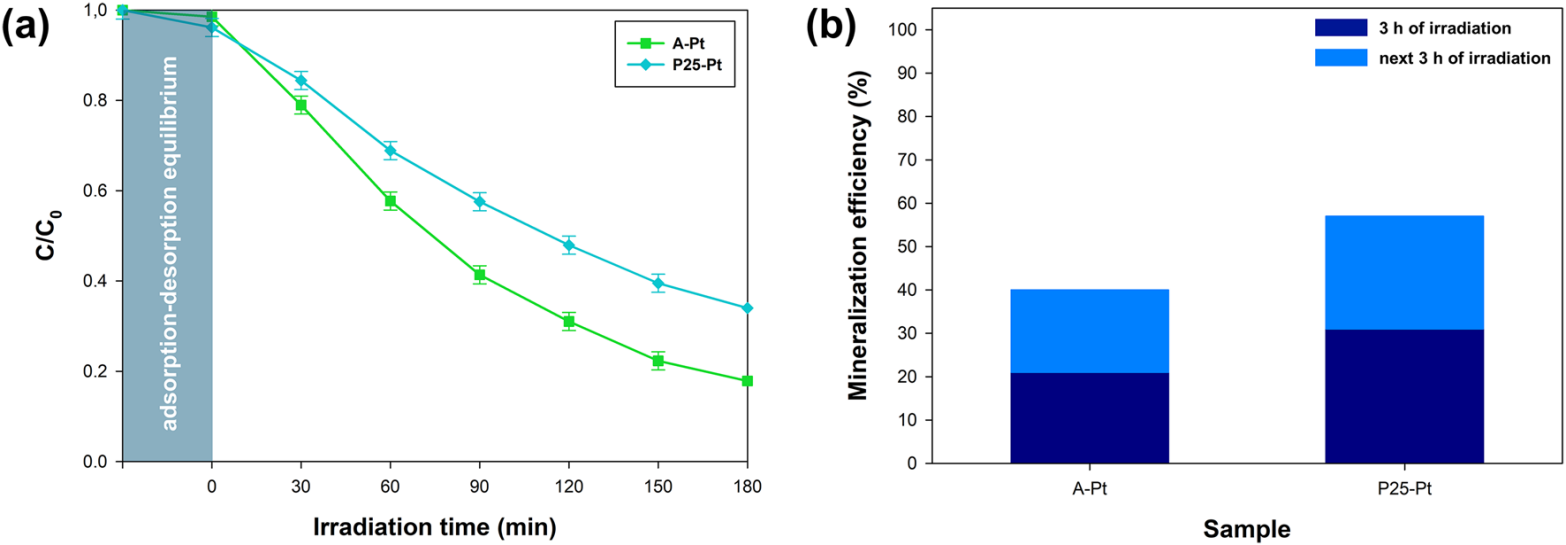
(**a**) photocatalytic degradation and (**b**) mineralization efficiency of NPX in sewage.

To evaluate the effectiveness of the developed photocatalysts, experiments were conducted using wastewater sourced from a municipal sewage treatment plant. LED light sources, tailored to match the optical characteristics of the synthesized photocatalysts, were employed for these tests with wastewater matrices. The results from these experiments are detailed in Fig. 8.

The collected data demonstrate the capability of the designed photocatalytic system to efficiently remove naproxen from wastewater. It is crucial to note that the choice of photocatalyst significantly influenced the process's effectiveness. The P25-Pt catalyst exhibited the highest removal rates, achieving over 80% degradation and more than 30% mineralization of naproxen after 180 min of exposure. In contrast, the A-Pt catalyst demonstrated approximately 65% removal efficiency for the same contaminant.

The decreased degradation performance of naproxen in sewage, compared to controlled laboratory solutions, is attributable to several complex factors within the wastewater matrix. Competing organic and inorganic substances, including suspended solids, compete for the active sites on the photocatalyst, thereby reducing the available sites for naproxen degradation. Moreover, natural organic matter in the wastewater can absorb reactive species like hydroxyl radicals, which are essential for breaking down pollutants, thereby reducing the overall efficiency of the process. Light absorption by colored and particulate materials in the sewage also hinders the activation of the photocatalyst. Over time, the intricate composition of the matrix may lead to catalyst poisoning, where ancillary substances clog the catalyst's active sites. Variations in ionic strength and pH levels may also impact the surface charge and stability of the catalyst. Despite these challenges, the photocatalytic system demonstrated a significant decrease in naproxen levels. Particularly noteworthy, mineralization rates after a prolonged period (6 h) approached 60%. Given the generally lower concentrations of pharmaceuticals in real environmental settings, these findings are encouraging and suggest substantial promise for the system's application in actual water treatment processes. This performance serves as a strong testament to the system's effectiveness and potential to enhance water quality.

## Conclusion

The primary objective of this investigation was to assess the influence of platinum nanoparticles on the photo-oxidative degradation of organic contaminants in the presence of TiO_2_. The study utilized advanced spectroscopic techniques to thoroughly analyze the interaction dynamics within TiO_2_-Pt systems under various LED illumination conditions. Notably, the findings challenge the notion that platinum nanoparticles extend the excitation spectrum of TiO_2_, suggesting instead that the observed photocatalytic enhancement is due to more efficient charge carrier separation, as evidenced by luminescence quenching data. The research underscored that UV-A irradiation does not lead to the formation of surface defects on TiO_2_, which has significant implications for the photodeposition process and the material's photocatalytic performance. The photooxidation experiments conducted, particularly with naproxen, demonstrated that the enhanced photocatalytic activity is not attributable to a widened excitation spectrum but rather to the effective separation of charge carriers facilitated by the platinum nanoparticles. Mass spectroscopy analyses further corroborated that the degradation pathways of naproxen are independent of the photocatalyst's physicochemical properties and the specific wavelengths used during photooxidation tests. This comprehensive approach, focusing on spectroscopic insights, provides a nuanced understanding of TiO_2_-Pt photocatalytic systems, offering valuable perspectives for future research in the field.

### Supplementary Information

Supplementary Information.

## Data Availability

The data that support the findings of this research are available from the corresponding author upon reasonable request.
