# Supplementary material for: Comprehensive spectroscopy and photocatalytic activity analysis of TiO2-Pt systems under LED irradiation
Source: Sci Rep. 2024 Jun 15;14:13827. doi: 10.1038/s41598-024-64748-4 (PMC11180208; doi:10.1038/s41598-024-64748-4)
Supplement: Supplementary file 1 — Supplementary Information. [file 41598_2024_64748_MOESM1_ESM.docx]

**Comprehensive spectroscopy and photocatalytic activity analysis of TiO_2_-Pt systems under LED irradiation**

Adam Kubiak^*^

*Adam Mickiewicz University, Poznan, Faculty of Chemistry, Uniwersytetu Poznanskiego 8, PL-61614 Poznan, Poland*

**Corresponding author: adam.kubiak@amu.edu.pl; Tel.: +48 61 829 17 21*


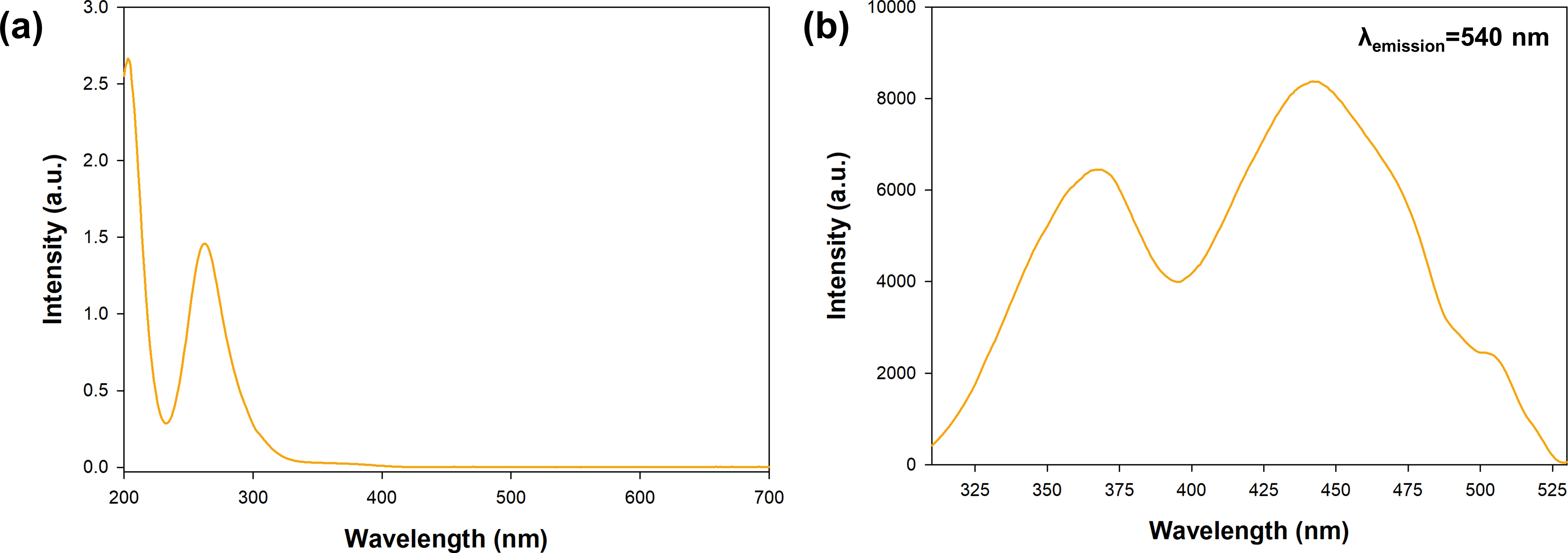


**Figure S1.** The (a) absorption and (b) excitation spectra of H_2_PtCl_6_.


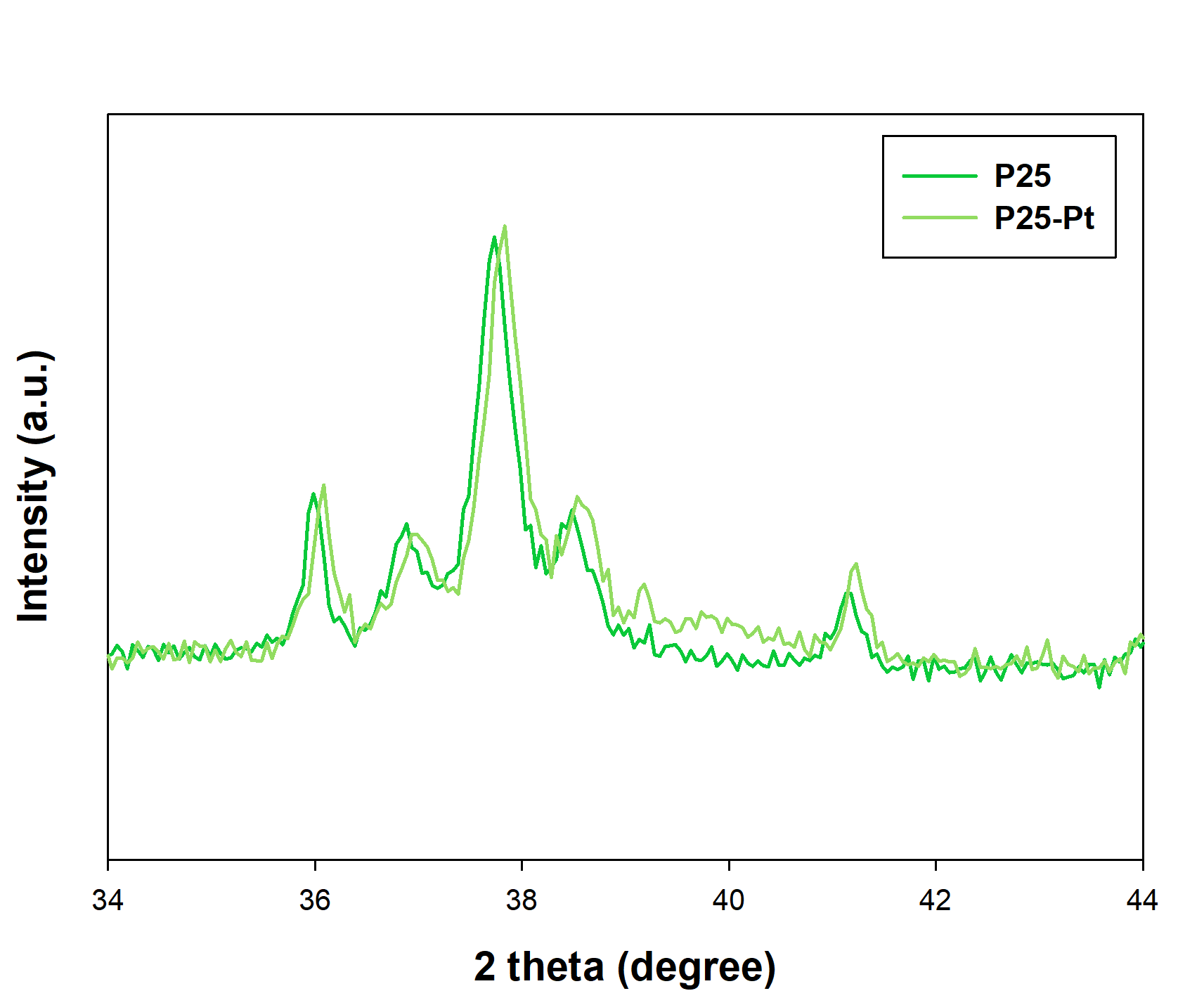


**Figure S2.** XRD pattern for P25-Pt sample in the range of 2θ=34-44 º


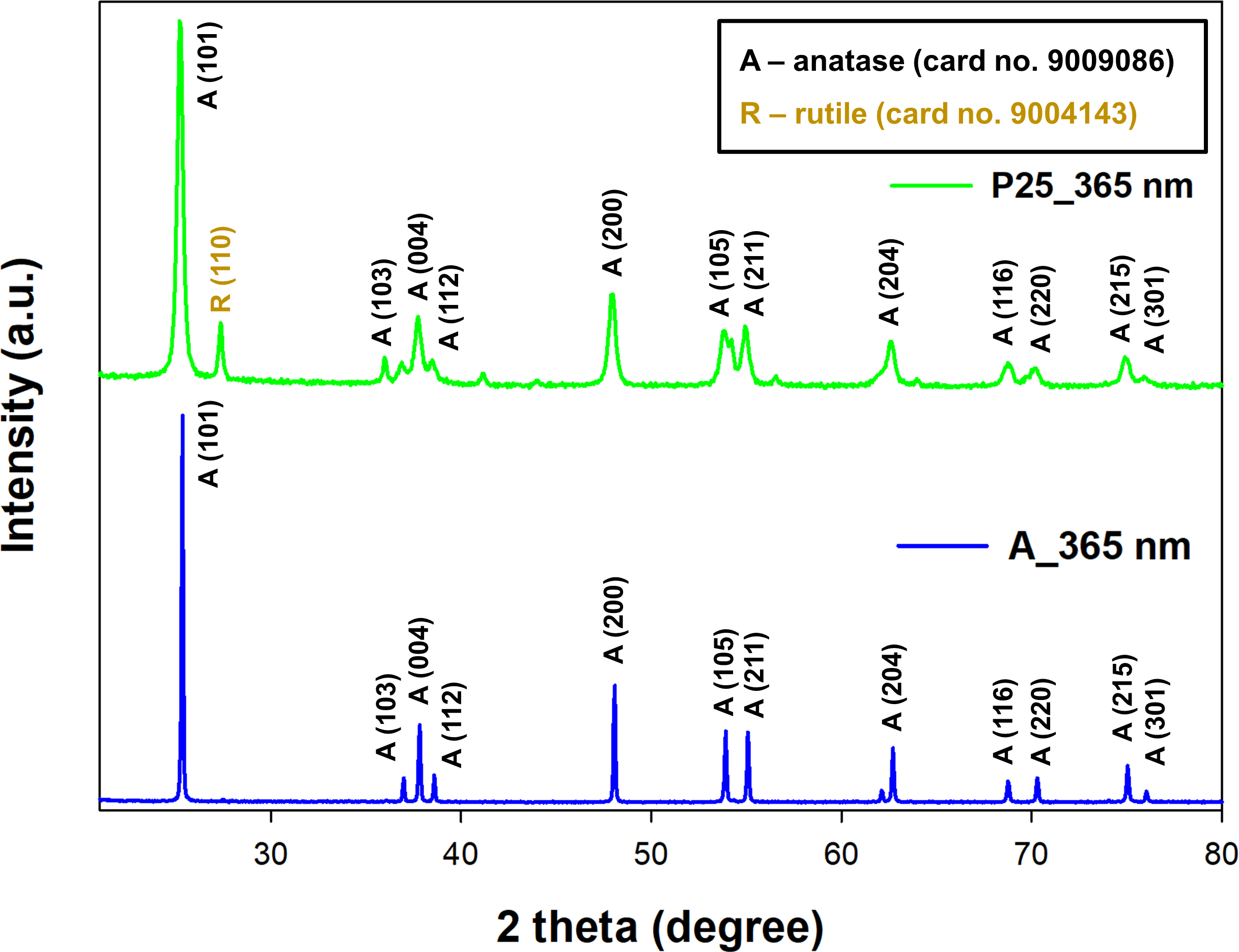


**Figure S3.** XRD patterns of reference TiO_2_ series samples.


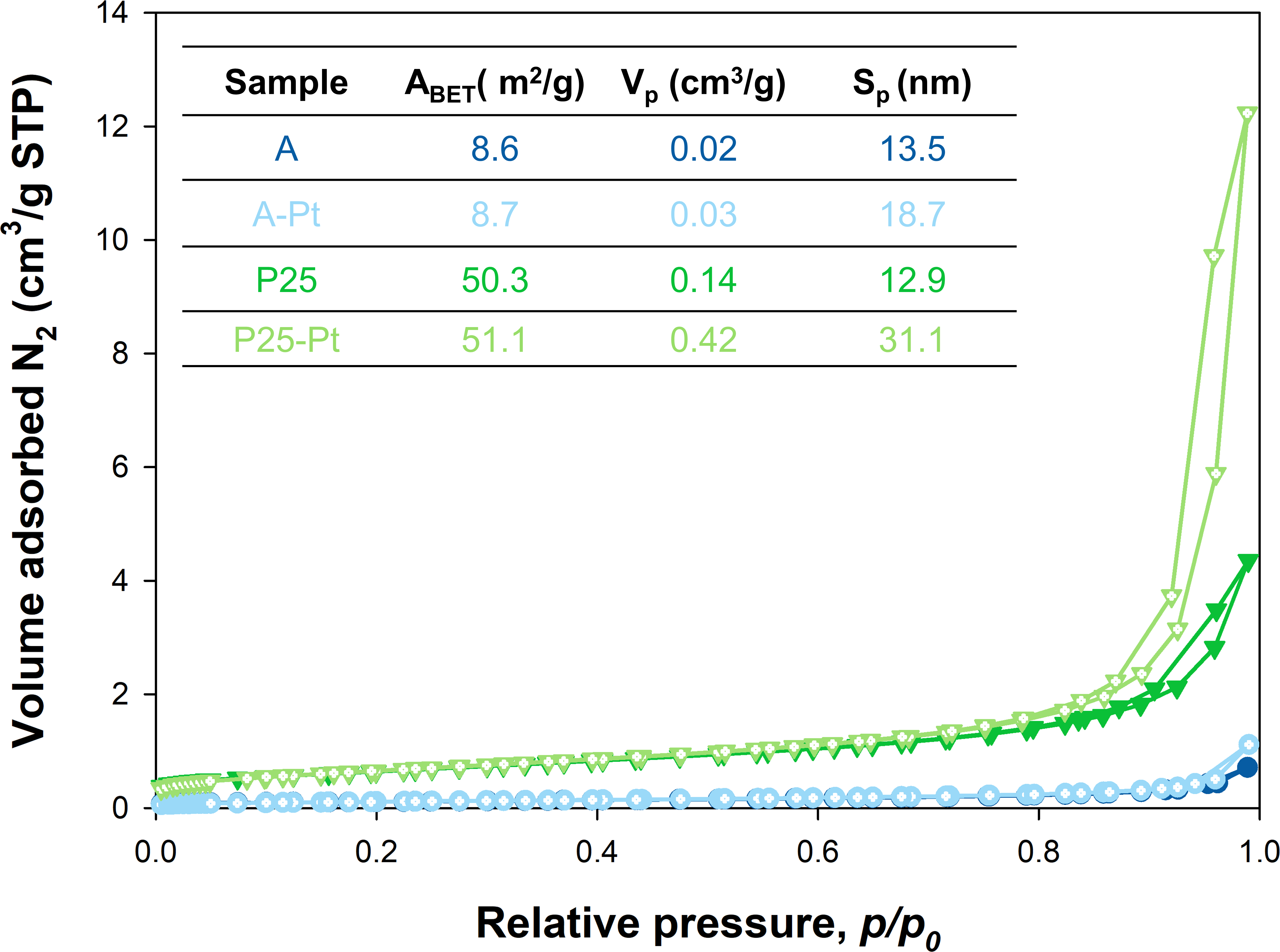


**Figure S4.** The isotherms of N_2_ sorption for TiO_2_-Pt systems and reference materials.


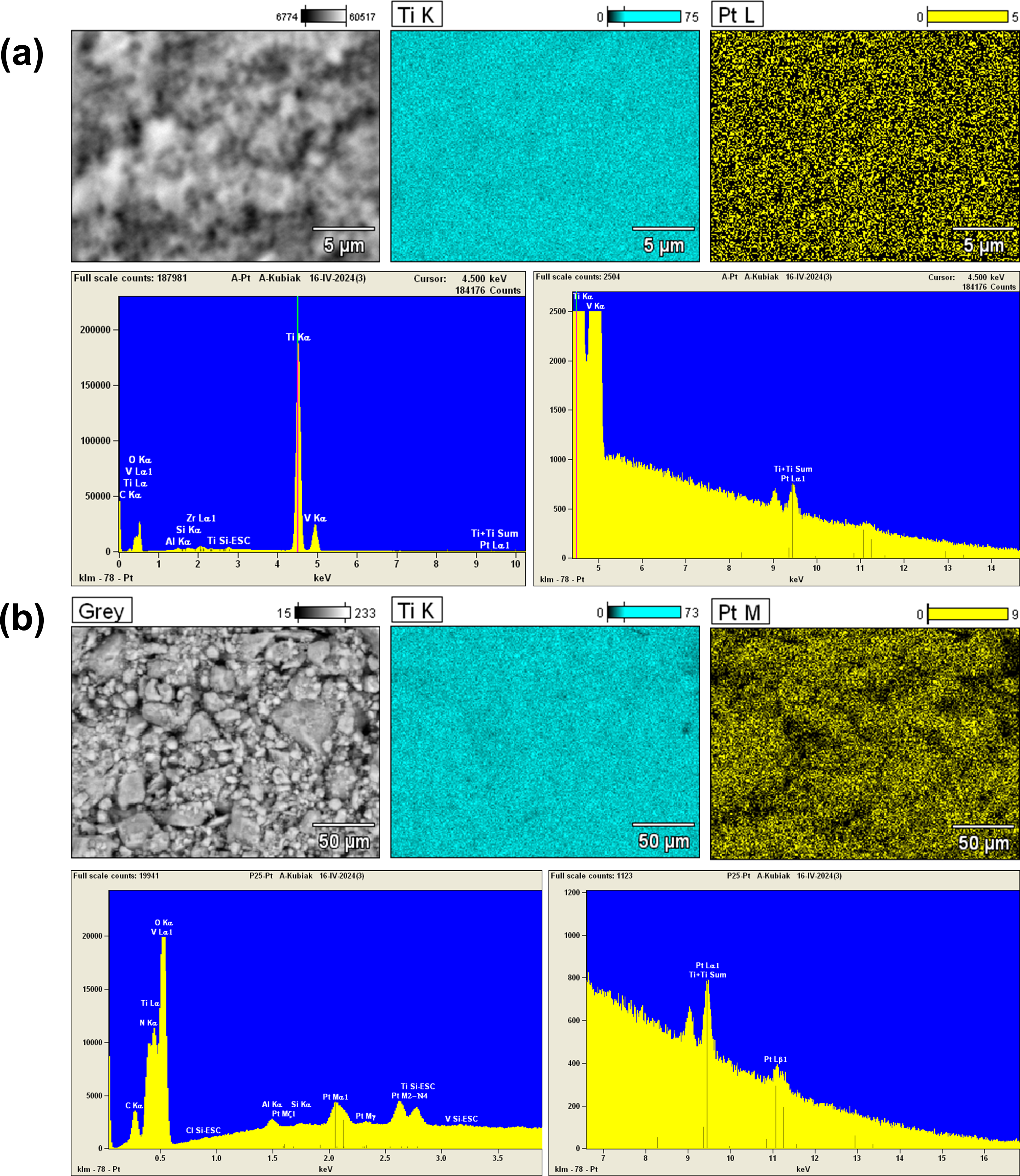


**Figure S5.** The results of EDX analysis for (a) A-Pt and (b) P25-Pt.


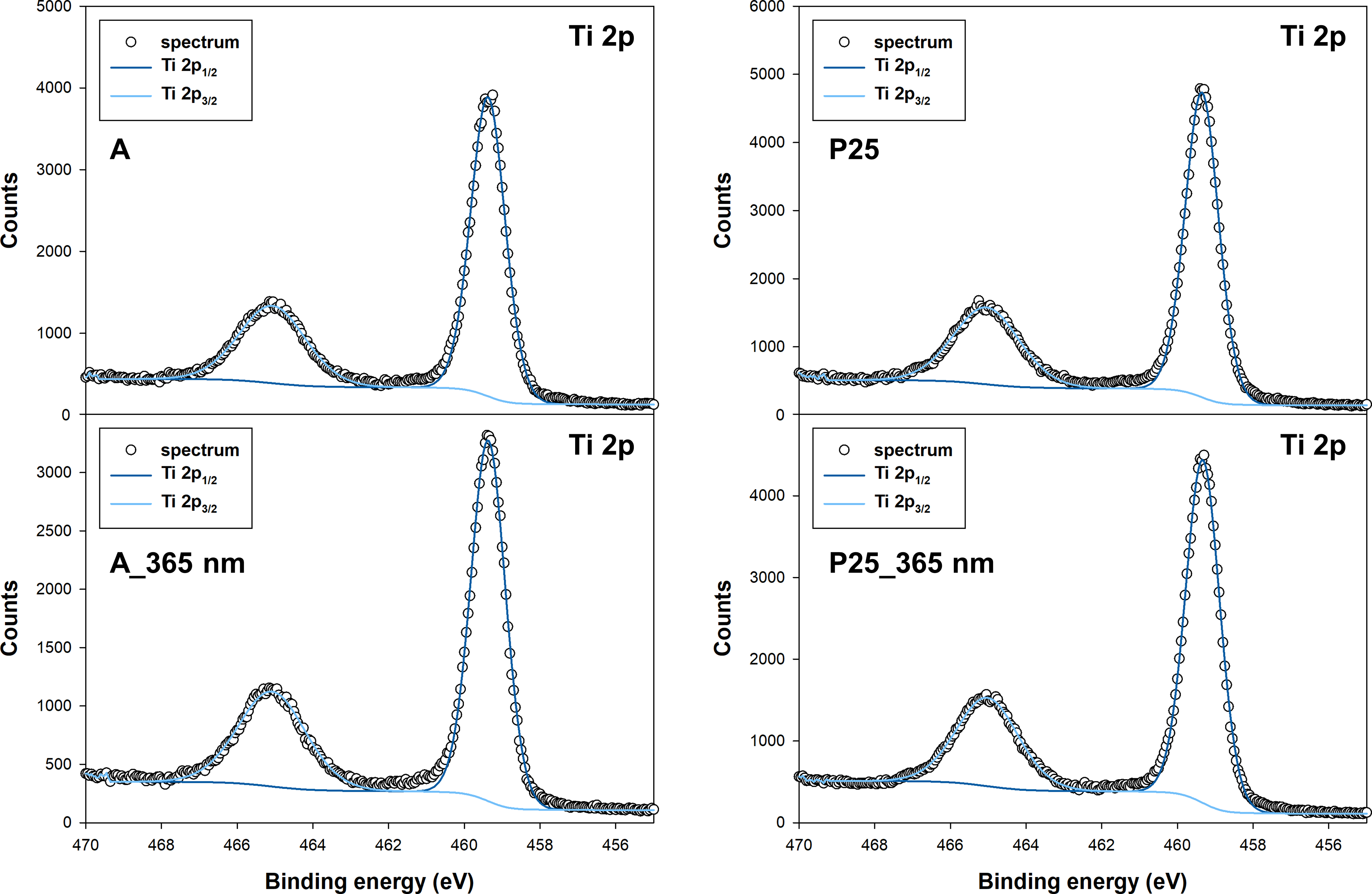


**Figure S6.** XPS analysis results for the bare TiO_2_ matrix and after exposure to UV-LED light.

**Table S1.** A cumulative list of platinum content determined by the EDXRF, AAS, and ICP-OES techniques.

| Analysis | Pt content |
| --- | --- |
| EDX | 1 wt.% |
| ICP-OES | 0.9 wt.% |
| AAS | 0.0307 Pt mg/L  (in the solution after the photo-deposition) |


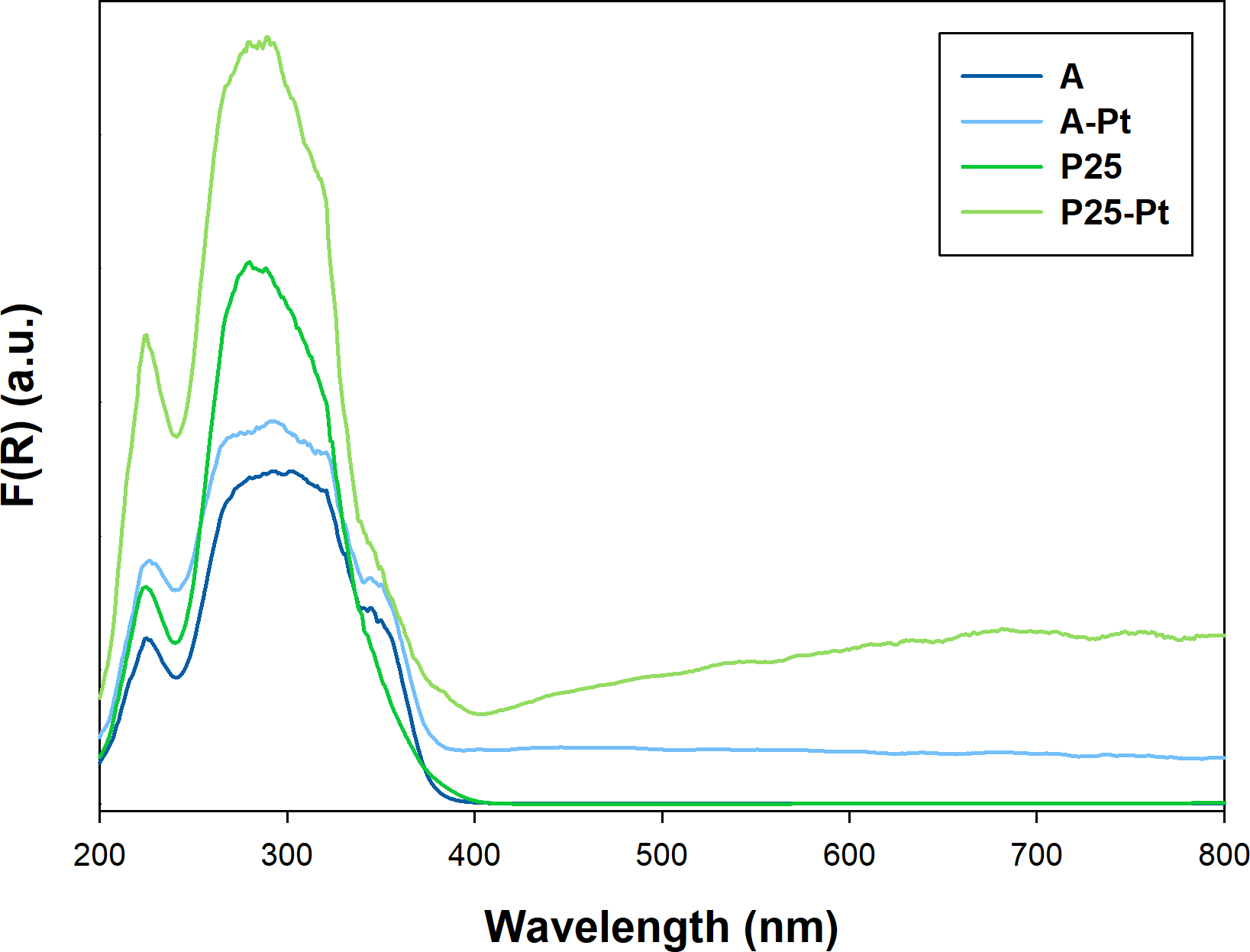


**Figure S7.** DRS spectra for TiO_2_-Pt materials synthesized using anatase and P25 as the TiO_2_ matrix.


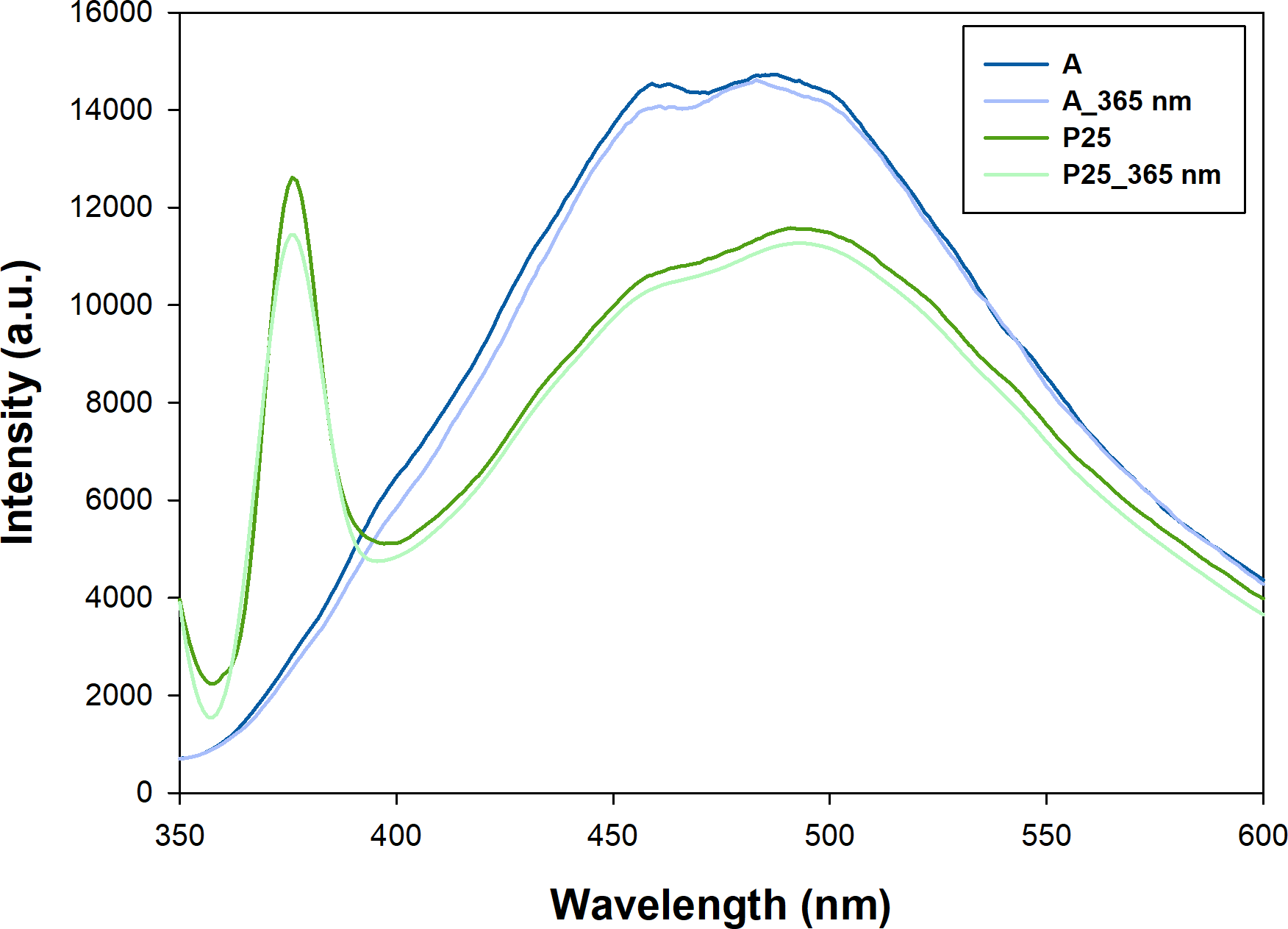


**Figure S8.** PL spectra of reference TiO_2_ series samples irradiated with LED light.


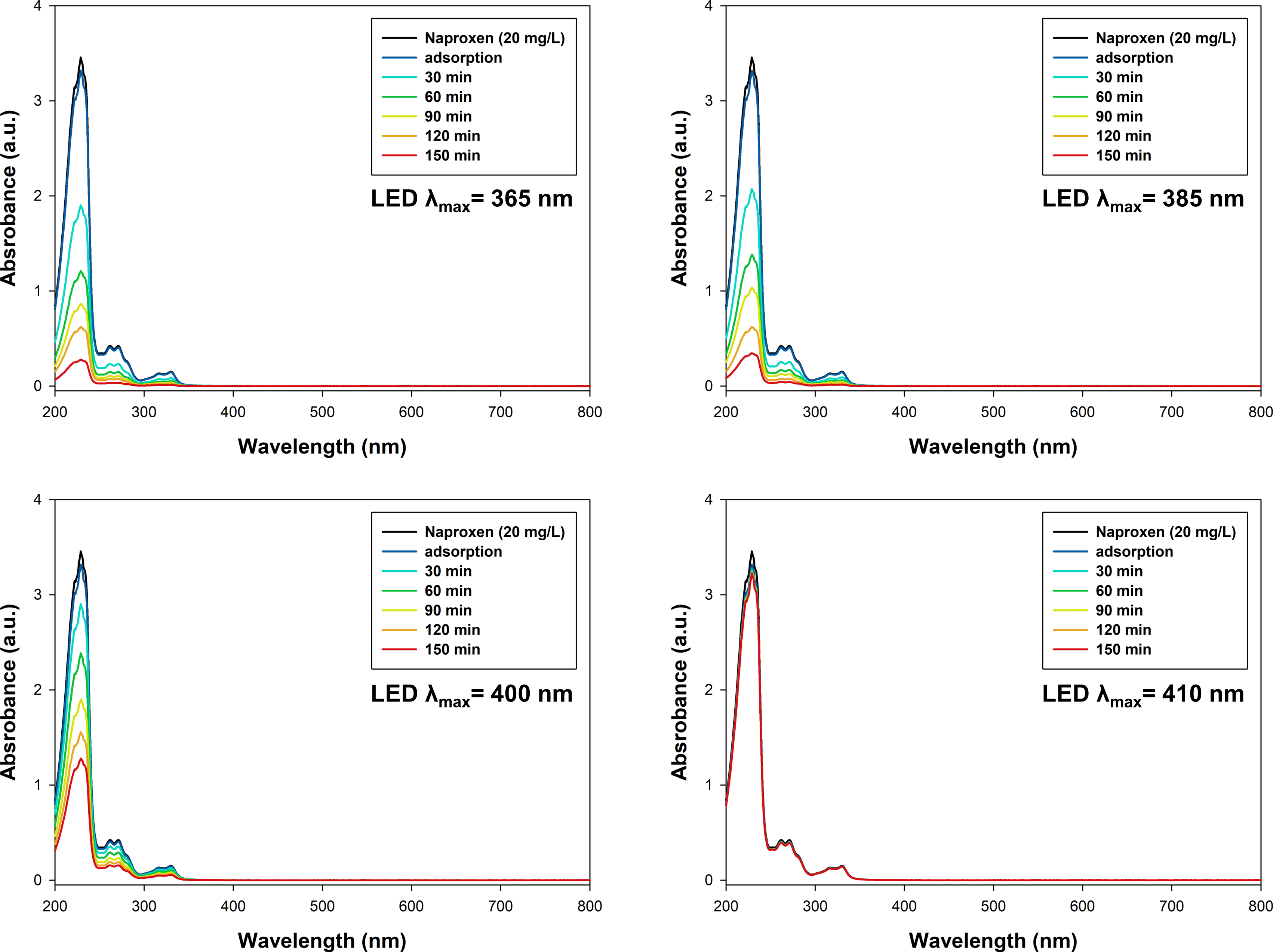


**Figure S9.** UV-Vis spectra of naproxen photodegradation using the A-Pt catalyst under various LED wavelength conditions.


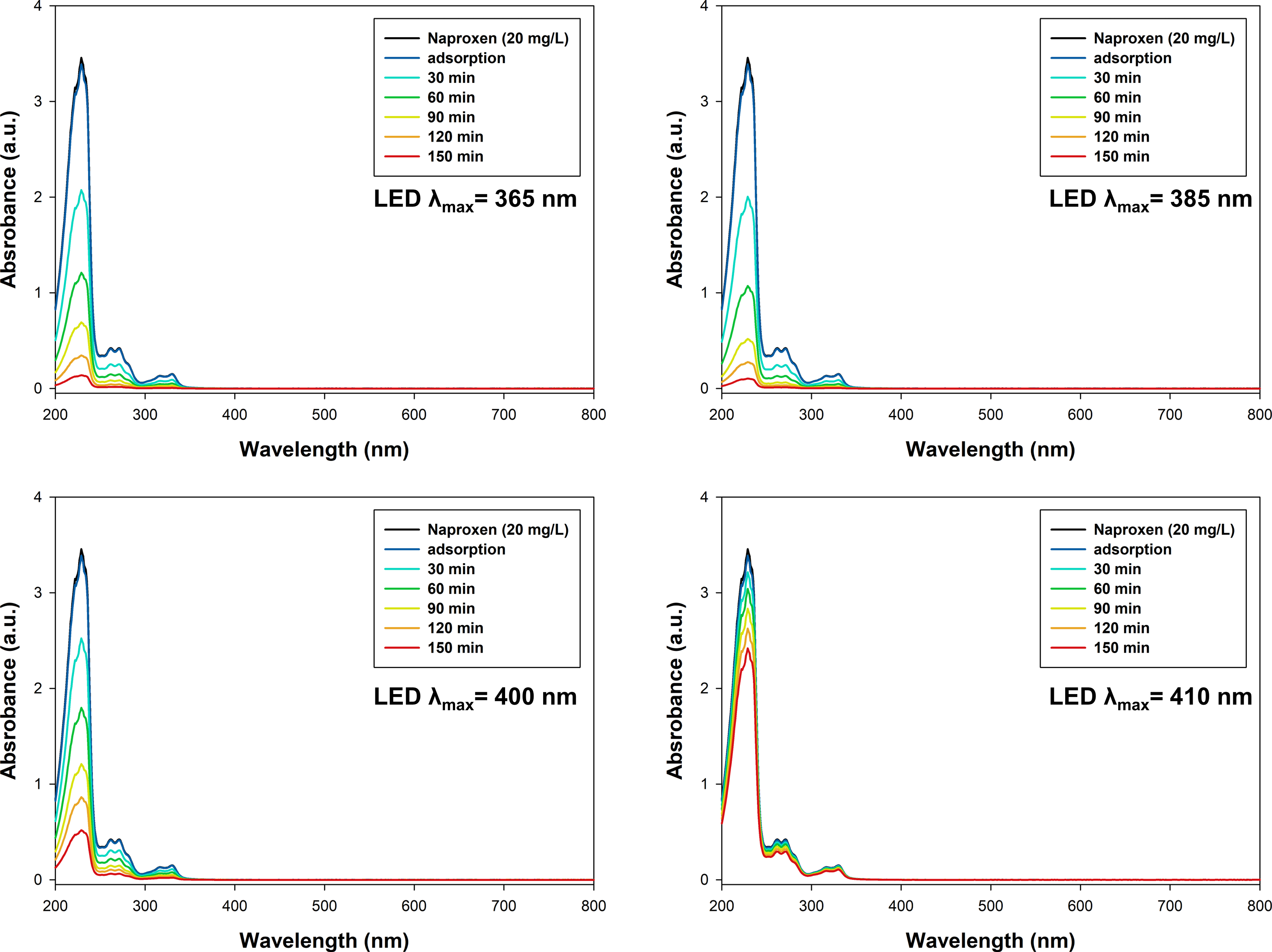


**Figure S10.** UV-Vis spectra of naproxen photodegradation using the P25-Pt catalyst under various LED wavelength conditions.


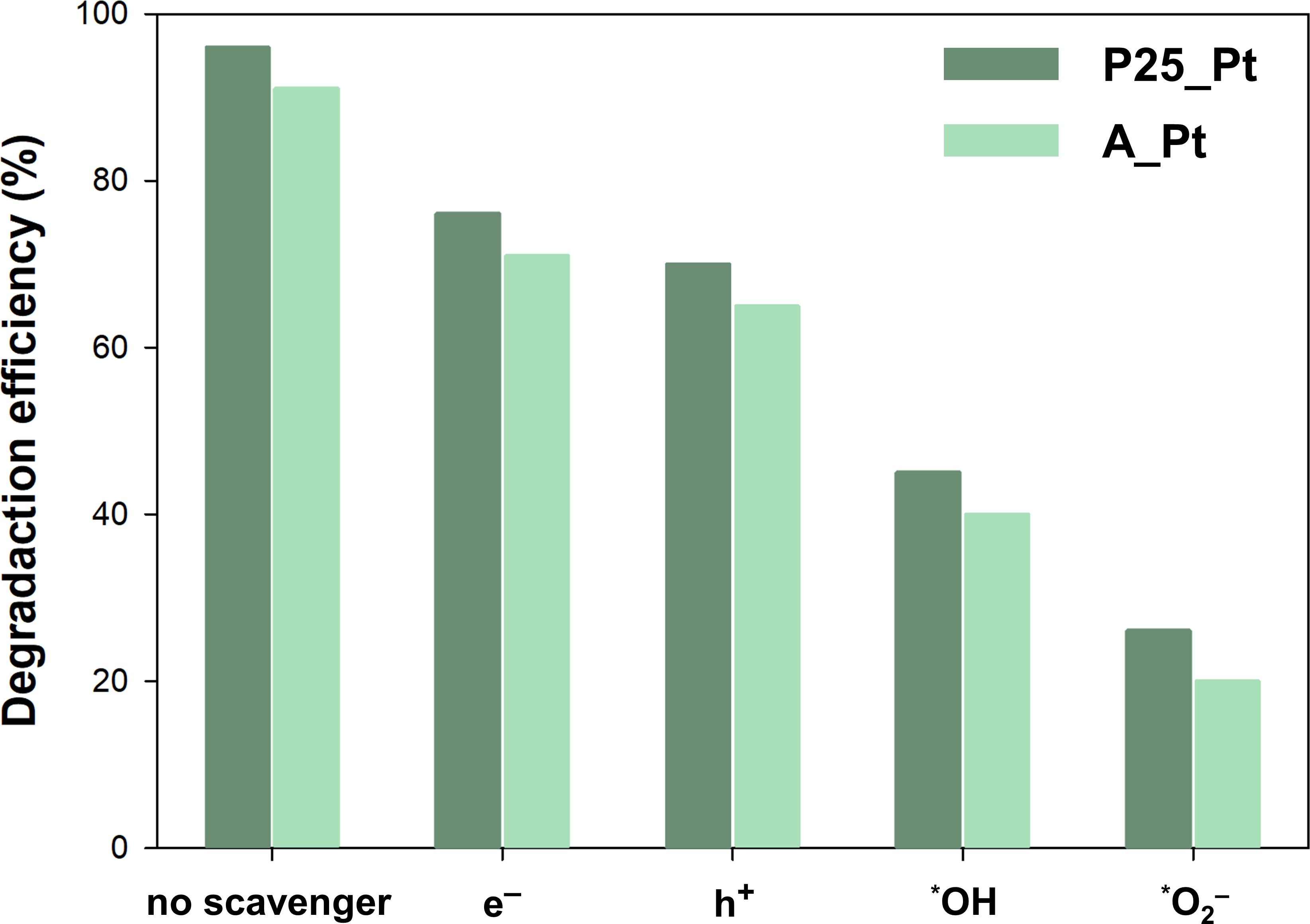


**Figure S11.** The degradation efficiency of NPX in the presence of radical scavengers.

**Table S2.** The calculated kinetic parameters for the photochemical degradation of naproxen.

| Sample | k_1_ (1/min) | (R^2^) |
| --- | --- | --- |
| anatase | | |
| A_365nm | 0.0071 | 0.998 |
| A_380nm | 0.0069 | 0.995 |
| A_400nm | 0.0033 | 0.995 |
| A_410nm | 0.0003 | 0.910 |
| A_430nm | 0.0003 | 0.924 |
| A_450nm | 0.0002 | 0.921 |
| A-Pt_365nm | 0.0145 | 0.998 |
| A-Pt_380nm | 0.0148 | 0.997 |
| A-Pt_400nm | 0.0058 | 0.998 |
| A-Pt_410nm | 0.0004 | 0.944 |
| A-Pt_430nm | 0.0004 | 0.917 |
| A-Pt_450nm | 0.0003 | 0.931 |
| P25 | | |
| P25_365nm | 0.0103 | 0.999 |
| P25_380nm | 0.0135 | 0.998 |
| P25_400nm | 0.0051 | 0.998 |
| P25_410nm | 0.0012 | 0.997 |
| P25_430nm | 0.0003 | 0.938 |
| P25_450nm | 0.0003 | 0.916 |
| P25-Pt_365nm | 0.0172 | 0.999 |
| P25-Pt_380nm | 0.0199 | 0.999 |
| P25-Pt_400nm | 0.0122 | 0.998 |
| P25-Pt_410nm | 0.002 | 0.998 |
| P25-Pt_430nm | 0.0003 | 0.911 |
| P25-Pt_450nm | 0.0003 | 0.924 |

**Table S3.** Detected intermediate products formed during the photooxidation of naproxen.

| Name | Mass (*m/z*) | Formula |
| --- | --- | --- |
| NPX | 230 | 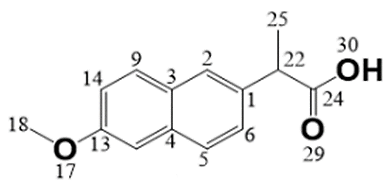 |
| TP1 | 185 | 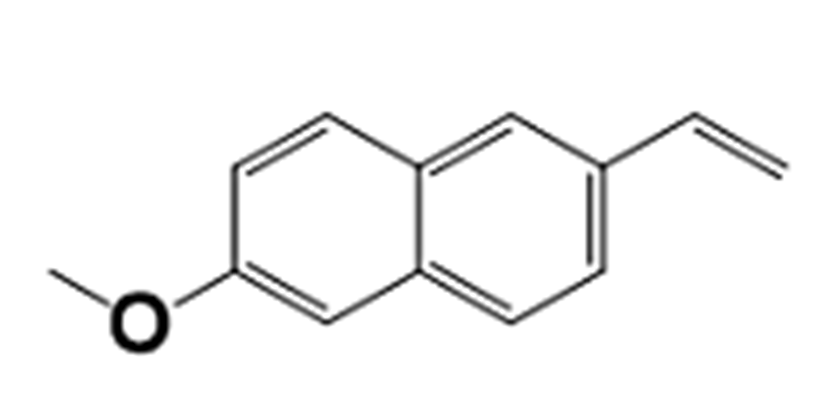 |
| TP2 | 201 | 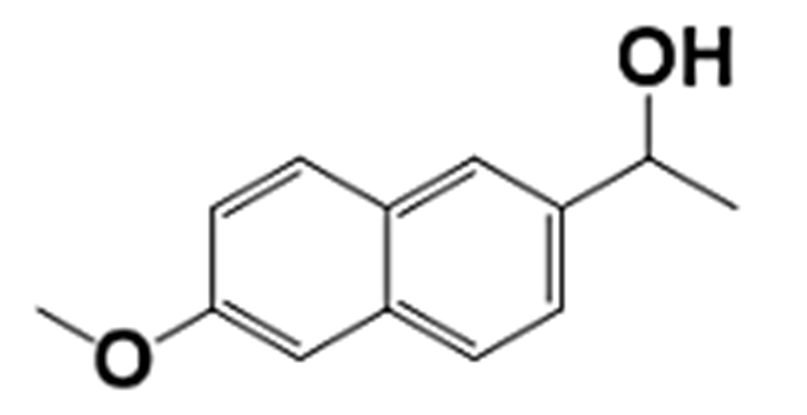 |
| TP3 | 223 | 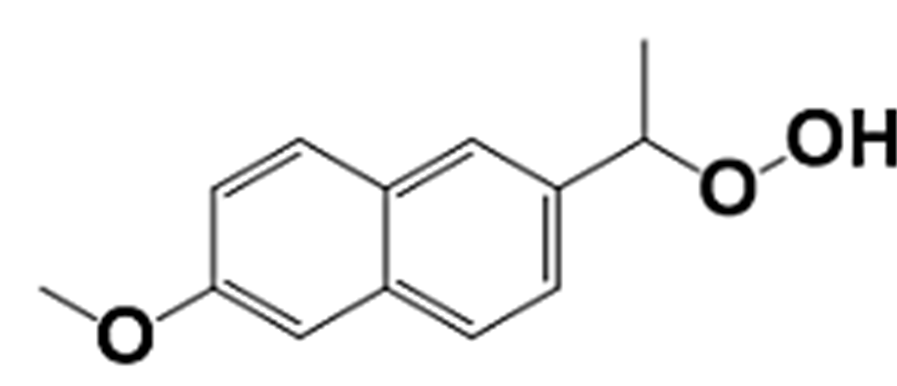 |
| TP4 | 158 | 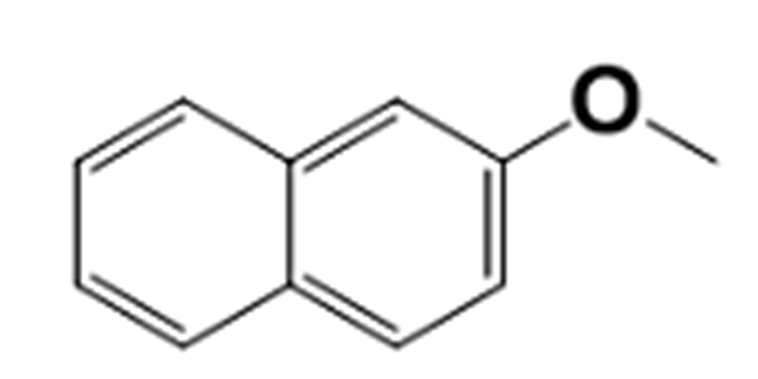 |
| TP5 | 134 | 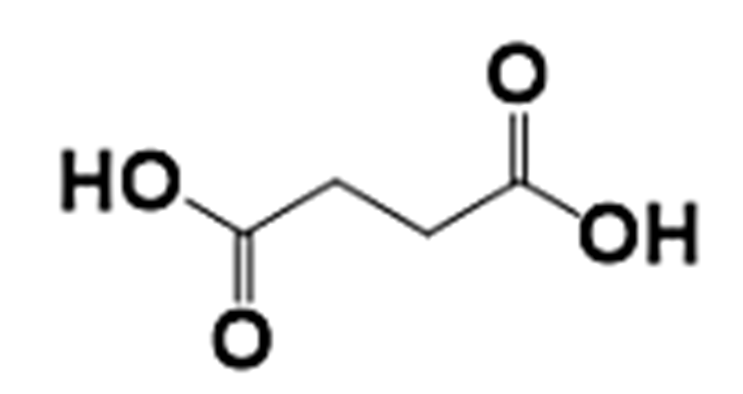 |
| TP6 | 148 | 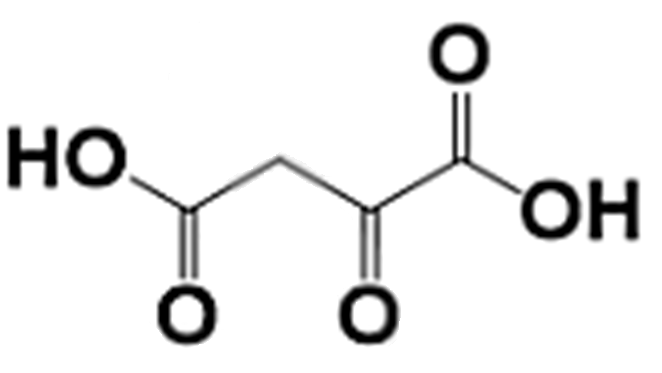 |

**Table S4.** The mass spectra of the generated ions during the photooxidation of NPX using TiO_2_-Pt photocatalysts

| NPX | *positive mode* |  |
| --- | --- | --- |
|  | *negative mode* |  |
| A-Pt_365nm  (60 min) | *positive mode* |  |
|  | *negative mode* |  |
| P25-Pt_365nm  (60 min) | *positive mode* |  |
|  | *negative mode* |  |
